# Impaired polyamine metabolism causes behavioral and neuroanatomical defects in a mouse model of Snyder–Robinson syndrome

**DOI:** 10.1242/dmm.050639

**Published:** 2024-05-09

**Authors:** Oluwaseun Akinyele, Anushe Munir, Marie A. Johnson, Megan S. Perez, Yuan Gao, Jackson R. Foley, Ashley Nwafor, Yijen Wu, Tracy Murray-Stewart, Robert A. Casero, Hülya Bayir, Dwi U. Kemaladewi

**Affiliations:** ^1^ Division of Genetic and Genomic Medicine, Department of Pediatrics, University of Pittsburgh School of Medicine, Pittsburgh, PA 15224, USA; ^2^ Department of Human Genetics, School of Public Health, University of Pittsburgh, Pittsburgh, PA 15261, USA; ^3^ Children's Neuroscience Institute, Department of Pediatrics, University of Pittsburgh School of Medicine, Pittsburgh, PA 15224, USA; ^4^ Sidney Kimmel Comprehensive Cancer Center, Johns Hopkins School of Medicine, Baltimore, MD 21224, USA; ^5^ Department of Developmental Biology, University of Pittsburgh School of Medicine, Pittsburgh, PA 15224, USA

**Keywords:** Spermine synthase, Spermine, Neurological functions, Rare disease, Pathogenesis, Mouse model

## Abstract

Snyder–Robinson syndrome (SRS) is a rare X-linked recessive disorder caused by a mutation in the *SMS* gene, which encodes spermine synthase, and aberrant polyamine metabolism. SRS is characterized by intellectual disability, thin habitus, seizure, low muscle tone/hypotonia and osteoporosis. Progress towards understanding and treating SRS requires a model that recapitulates human gene variants and disease presentations. Here, we evaluated molecular and neurological presentations in the G56S mouse model, which carries a missense mutation in the *Sms* gene. The lack of SMS protein in the G56S mice resulted in increased spermidine/spermine ratio, failure to thrive, short stature and reduced bone density. They showed impaired learning capacity, increased anxiety, reduced mobility and heightened fear responses, accompanied by reduced total and regional brain volumes. Furthermore, impaired mitochondrial oxidative phosphorylation was evident in G56S cerebral cortex, G56S fibroblasts and *Sms-*null hippocampal cells, indicating that SMS may serve as a future therapeutic target. Collectively, our study establishes the suitability of the G56S mice as a preclinical model for SRS and provides a set of molecular and functional outcome measures that can be used to evaluate therapeutic interventions for SRS.

## Teaser

**Summary:** Characterization of a mouse model of Snyder–Robinson syndrome reveals impaired neurological functions and mitochondrial respirations and provides a set of outcome measures to evaluate future therapeutic interventions.

**Research Simplified**

Snyder–Robinson syndrome (SRS) is a very rare genetic disorder characterised by intellectual disability, muscular and skeletal abnormalities, thin body build, reduced bone density and cognitive impairments that mostly affect males. Individuals carrying variants of a gene that codes for the protein spermine synthase – important for production of metabolites called polyamines that regulate critical cellular functions – often have SRS. Gaining a better understanding of the cause and effects of this disease in an animal model can help researchers design therapeutics for SRS.

In this study, the authors generated a laboratory mouse model by mutating the gene that codes for spermine synthase. They found that spermine synthase was almost completely absent in the brain and skeletal muscles of these mice, which was accompanied by thin body build, shortened stature, low bone density and failure to survive healthily – all symptoms of SRS in humans. Furthermore, these mice had elevated anxiety responses and cognitive impairment, possibly due to a reduced brain size, which is associated with SRS in humans.

This study reveals that the symptoms of SRS can be recapitulated in this laboratory mouse model. Further research using this model can help better understand disease progression of spermine synthase-based SRS, which can facilitate development of effective therapies for SRS.

## INTRODUCTION

The polyamines putrescine, spermidine and spermine are positively charged metabolites involved in various cellular functions, such as maintaining chromatin structure, regulating gene expression and fine-tuning metabolic pathways. They are also crucial for immune cell activation, wound healing, tissue growth and development (Bachrach et al., 2001; Landau et al., 2010; Hesterberg et al., 2018; Kemaladewi et al., 2018; Akinyele and Wallace, 2022; Arruabarrena-Aristorena et al., 2018; Holbert et al., 2022). The levels of intracellular polyamines are tightly regulated by *de novo* synthesis, interconversion and transport.

**Fig. 1.**
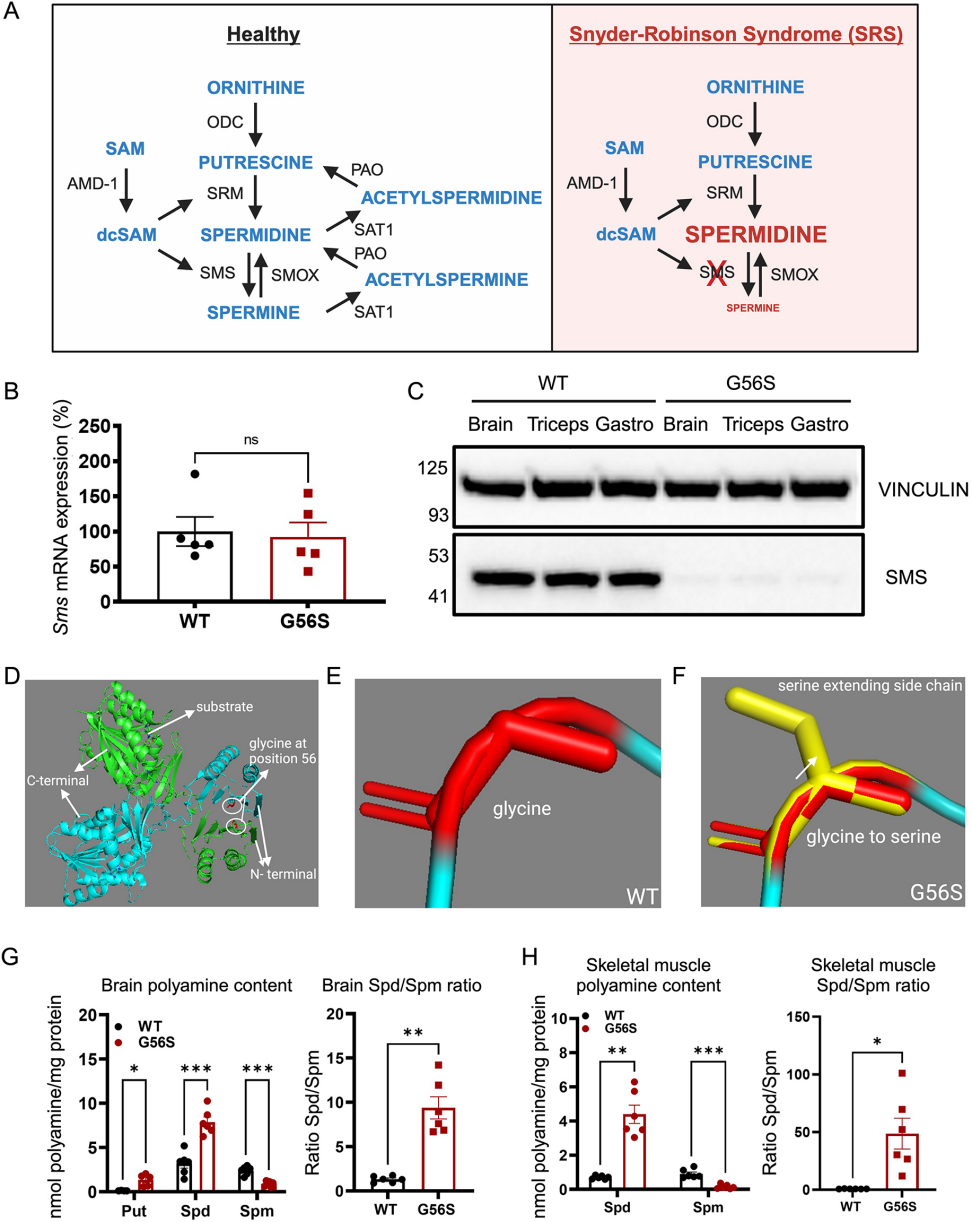
**Lack of spermine synthase and perturbation of polyamine metabolism in SRS.** (A) Polyamine metabolism pathway in humans with SRS compared with unaffected individuals. The absence of SMS leads to a decrease in the conversion of spermidine to spermine, leading to increased spermidine and decreased spermine content. AMD-1, adenosylmethionine decarboxylase; dcSAM, decarboxylated s-adenosylmethionine; ODC, ornithine decarboxylase; PAO, acetylpolyamine oxidase; SAM, s-adenosylmethionine; SAT1, spermidine/spermine acetyltransferase; SMOX, spermine oxidase; SMS, spermine synthase; SRM, spermidine synthase. (B) *Sms* mRNA expression in the brain of 7-week-old wild-type (WT) and G56S mutant mice. (C) SMS protein expression in brain and skeletal muscles (triceps and gastrocnemius) of 7-week-old wild-type and G56S mice. (D) 2D-crystal structure of the SMS protein with glycine at position 56 in the N-terminal region (circled). The 2D-crystal structure of SMS was modeled from protein data bank ID: 3C6M. (E) The atomic structure of glycine at position 56 in the N-terminal region of SMS protein. (F) Serine in place of glycine at position 56 of SMS protein; the extended serine sidechain is highlighted in yellow. (G,H) Brain (G) and skeletal muscle (H) polyamine content and spermidine/spermine (Spd/Spm) ratios in 24-week-old wild-type and G56S mice quantified by HPLC. Note that putrescine levels were below the limit of detection in the G56S skeletal muscle. Data represent mean±s.e.m. from *n*=3-5 mice per group. ns, not significant. *P<0.05, **P<0.01, ***P<0.001 (unpaired two-tailed *t*-tests).

Putrescine is the main precursor in the *de novo* synthesis pathway, leading to the formation of the higher-order polyamines spermidine and spermine (Fig. 1A). These reactions are controlled by a set of enzymes, namely spermidine synthase (SRM) and spermine synthase (SMS). Dysregulation or lack of the enzymes involved in this process can cause aberrations in polyamine metabolism, thereby contributing to underlying disease conditions, such as cancers, Alzheimer's disease and Snyder–Robinson syndrome (SRS) (Lewandowski et al., 2010; Soda, 2011; Milovic and Turchanowa, 2003; Schwartz et al., 2013). [Boxed-text DMM050639B1]

SRS (OMIM: 309583) is a rare X-linked intellectual disability syndrome associated with pathogenic variants in the *SMS* gene that lead to the loss or reduction of SMS enzymatic activity (Milovic and Turchanowa, 2003) (Fig. 1A). Consequently, the level of spermidine is elevated, whereas spermine is reduced. Individuals with SRS have altered spermidine/spermine ratio and exhibit thin body habitus, low muscle tone, developmental delays and seizures (Schwartz et al., 2013; de Alencastro et al., 2008; Larcher et al., 2020), which worsen over time (Schwartz et al., 2013; Valera Ribera et al., 2022). Some individuals with SRS also have difficulty with walking, and some never achieve ambulation (de Alencastro et al., 2008). Although mutation in the *SMS* gene was identified as the cause of SRS as early as 2003 (Cason et al., 2003), no suitable mammalian model exists to study disease pathophysiology and to develop effective therapeutic interventions. Previously, a mouse model called *Gy* (Gyro; because of its circling behavior) (Wang and Pegg, 2011) harboring a complete deletion of the *Sms* gene was used to study SRS pathophysiology (Meyer et al., 1998; Lyon et al., 1986). However, the presence of an additional mutation in the *Phex* gene, encoding phosphate-regulating endopeptidase homolog, which is involved in phosphate transport and causes bone-related diseases (Meyer et al., 1998; Lyon et al., 1986), complicated the interpretation of many of the abnormalities observed in this mouse model.

Here, we describe the disease presentation in a recently generated mouse model of SRS (https://www.jax.org/news-and-insights/2019/july/new-research-models-for-snyder-robinson-syndrome) that carries a mutation variant analogous to that reported in individuals diagnosed with severe SRS (de Alencastro et al., 2008). Our findings revealed that the mutant mice recapitulated many phenotypic defects characteristics of SRS, including failure to thrive, short stature, decreased bone density, cognitive impairments and reduced brain volumes. Furthermore, transcriptomic analysis and functional assay in various SMS-deficient models identified impaired mitochondrial oxidative phosphorylation as one of the molecular mechanisms underlying SRS pathogenesis.

## RESULTS

### Loss of SMS expression alters polyamine content in mice

A mouse model carrying a missense mutation in the *Sms* gene was generated in a collaborative effort between the Snyder–Robinson Syndrome Foundation and the Jackson Laboratory Rare Disease Translational Center (https://www.jax.org/news-and-insights/2019/july/new-research-models-for-snyder-robinson-syndrome). Specifically, the mice, hereafter called G56S mice, harbor two nucleotide changes (GGC to TCC) in exon two of the *Sms* gene, resulting in a glycine-to-serine substitution at position 56 of the SMS protein.

We first determined whether the G56S mutation altered the SMS expression profile and tissue polyamine levels. We found that the level of *Sms* mRNA remains unchanged (Fig. 1B). However, there was a near-complete loss of SMS protein in both the brain and the skeletal muscles of G56S mice (Fig. 1C).

To further understand the impact of the mutation on SMS structure and functions, we performed in silico 2D modeling to visualize the C-terminal and the N-terminal domains, which are important for catalytic activity and dimerization, respectively (Fig. 1D). Compared with the wild-type SMS (Fig. 1E), the presence of serine at position 56 in the G56S SMS mutant creates an extended side chain which that interferes with monomer dimerization (Fig. 1F), as previously described by Zhang et al. (2010), and may account for the loss of SMS protein expression despite the normal transcript level.

Consequently, the spermidine level was elevated and the spermine level was reduced, resulting in a significantly higher spermidine/spermine ratio in the G56S brain (Fig. 1G) and skeletal muscles (Fig. 1H). The putrescine level was also increased in the G56S brain (Fig. 1G); however, it fell below the detection limit in the skeletal muscles. Overall, the loss of SMS expression in G56S mice resulted in elevated spermidine and ratio of spermidine/spermine, similar to that described in cells from patients with SRS (Stewart et al., 2023).

### Biometric parameters are significantly altered in G56S mice

**Fig. 2.**
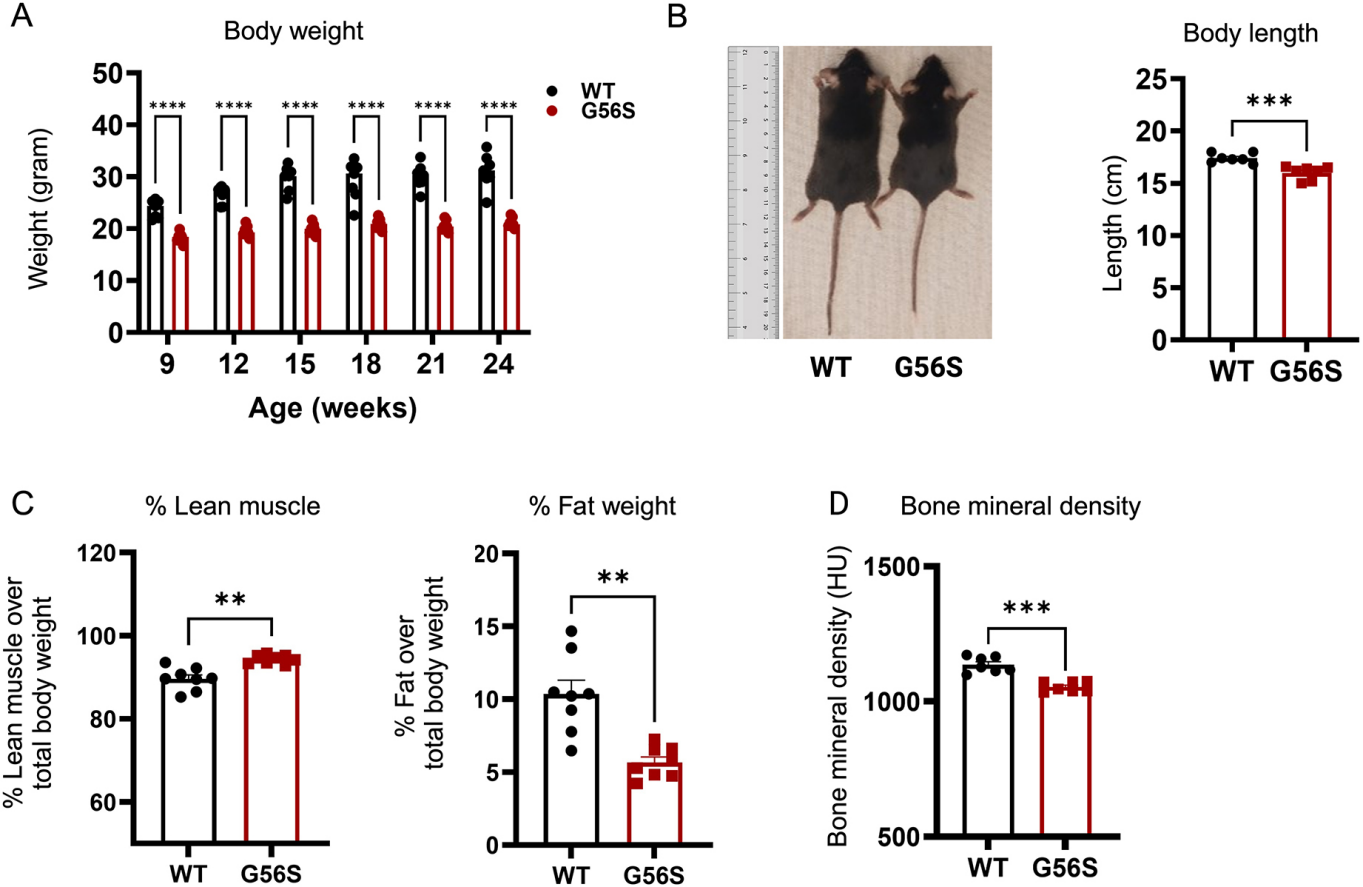
**Biometric analyses of G56S and wild-type mice.** (A) Body weight was measured at the indicated ages. (B) Body length of 24-week-old mice. (C) Body composition of 15-week-old mice (percentage lean muscle and percentage fat weight) determined by EchoMRI scan. (D) Bone mineral density of 20-week-old mice measured by micro-CT scan. Data represent mean±s.e.m., *n*=7 mice per group. **P<0.01, ***P<0.001, ****P<0.0001 (unpaired two-tailed *t*-tests). WT, wild type.

SRS-affected individuals typically exhibit an asthenic physique with a thin body build, short stature, low muscle tone and failure to thrive (Schwartz et al., 2013). Therefore, we interrogated whether some of these features are present in the G56S mice. We observed that the G56S mice have significantly lower body weight (Fig. 2A) and reduced length (Fig. 2B) compared with the age-matched wild-type counterparts. We found no significant differences in the amount of food consumed by the wild-type mice and the G56S mice, as measured using a comprehensive laboratory animal monitoring system (CLAMS) (Nie et al., 2015; Wang et al., 2009) (Fig. S1A), suggesting that the failure to thrive is attributed to the disease and not food intake.

Subsequently, we analyzed the body composition of the mice using EchoMRI. We found that the G56S mice had a higher percentage of lean muscle mass (Fig. 2C). Further examination of the muscle phenotypes revealed no difference in muscle fiber size (Fig. S1B,C) and grip strength (Fig. S2A) between the wild-type and G56S mutant mice. Interestingly, despite the lack of apparent muscle phenotypes, there was a significant reduction in the fat weight in the G56S mice compared with their wild-type counterparts (Fig. 2C), reflecting the asthenic feature of individuals with SRS.

Next, we assessed whether bone deformities were present in the G56S mice. We subjected the mice to whole-body 3D microcomputed tomography (micro-CT) imaging and found that the G56S mice had significantly lower bone mineral density (Fig. 2D, Fig. S2B). Collectively, altered biometric readouts, such as shortened stature, failure to thrive, reduced body fat and bone mineral density, indicate that polyamine perturbation impacts the growth and development in the G56S mice.

### G56S mice exhibit less activity and exploratory behavior than the wild type

**Fig. 3.**
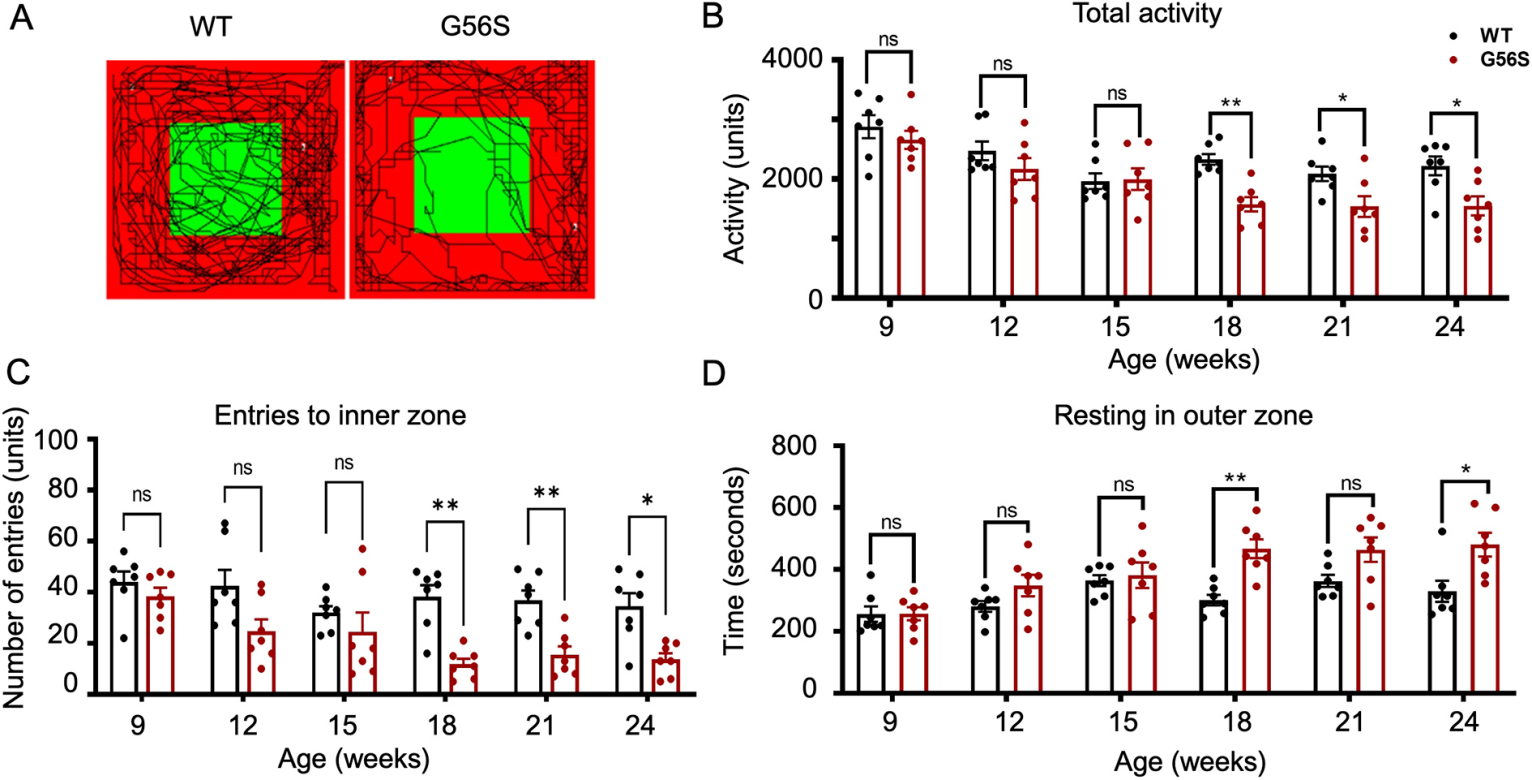
**Anxiety-related response monitoring in an open-field test.** (A) Representative movement pattern of 24-week-old wild-type and G56S mice. Green, inner zone; red, outer zone. (B) Total activity of the animals at the indicated ages. (C) Number of entries to the inner zone of the open-field chamber. (D) Resting time (seconds) in the outer zone of the open-field chamber. Data represent mean±s.e.m., *n*=7. *P<0.05; **P<0.01 (unpaired two-tailed *t*-tests). ns, not significant. WT, wild type.

To assess their neurological presentations, the mice were subjected to a longitudinal open-field test, which gauges general locomotion and anxiety-like behavior over time. We observed a significant reduction in the total activity of the G56S mice starting at the age of 18 weeks old (Fig. 3A,B). The G56S mice also demonstrated a lack of exploratory behavior, as shown by their reluctance to enter the inner zone (Fig. 3C) and tendency to stay in the outer zone (Fig. 3D) of the open-field arena. Such behavior is indicative of an anxiety-related response, which is one of the prominent features of neurological disorders such as SRS. Interestingly, there was no difference in the activity and exploratory behavior between G56S and wild-type animals younger than 18 weeks old, suggesting a progressive nature of the disease.

### Cognitive impairment is evident in G56S mice

**Fig. 4.**
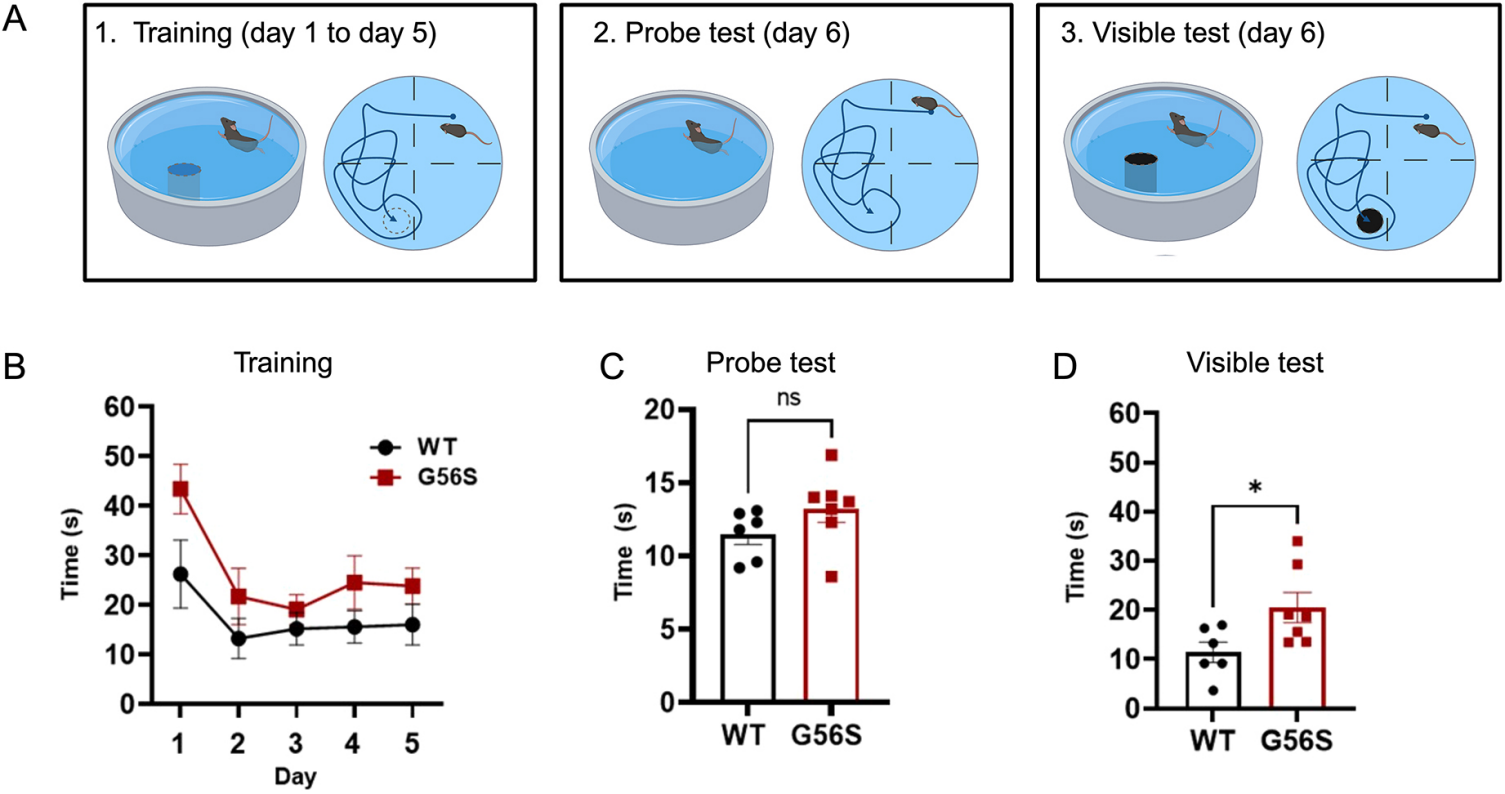
**Performance of 16-week-old G56S and wild-type mice in the MWM test.** (A) Illustration of the three components of the MWM test. The escape platform is submerged/hidden during the training period (daily for 5 days), removed during the probe test (day 6), or placed above the water level during the visible test (day 6). The top view of the area illustrates the location of the quadrants in which the animals are placed and the placement of the escape platform. (B) Time required to locate a hidden/submerged escape platform on each day of the five-day training period. (C) Time spent in the escape quadrant during the probe test, in which the platform was absent. (D) Time required to locate a visible escape platform on day 6. Data represent mean±s.e.m., *n*=7 mice per group. *P<0.05 (two-way ANOVA for repeated measures for B and unpaired two-tailed *t*-tests for C,D). ns, not significant. WT, wild type.

One of the major neurological presentations in SRS is mild to severe cognitive impairments (de Alencastro et al., 2008). Therefore, we performed a Morris water maze (MWM) assay to assess spatial memory and learning in the G56S mice (Fig. 4A). First, the animals were trained daily to navigate and locate an escape platform that was submerged or hidden under the water. The time required for the animals to reach the escape quadrant was recorded. On day six, the platform was either removed (probe test) or placed above the water level (visible test).

We observed that, in general, the G56S mice took longer to find the escape quadrant compared with their wild-type counterparts. The trends were not significant during the training period (Fig. 4B) and in the probe test (Fig. 4C). However, there was a significant difference in the visible test (Fig. 4D). These data indicate that the G56S mice were less effective in retaining the information necessary to complete the task compared with the wild-type counterparts, suggesting some degree of learning impairments. It is important to emphasize that the mice were only tested at 16 weeks. Thus, we were unable to verify any age-related decline in the learning impairments.

### The G56S mice demonstrate heightened fear responses

**Fig. 5.**
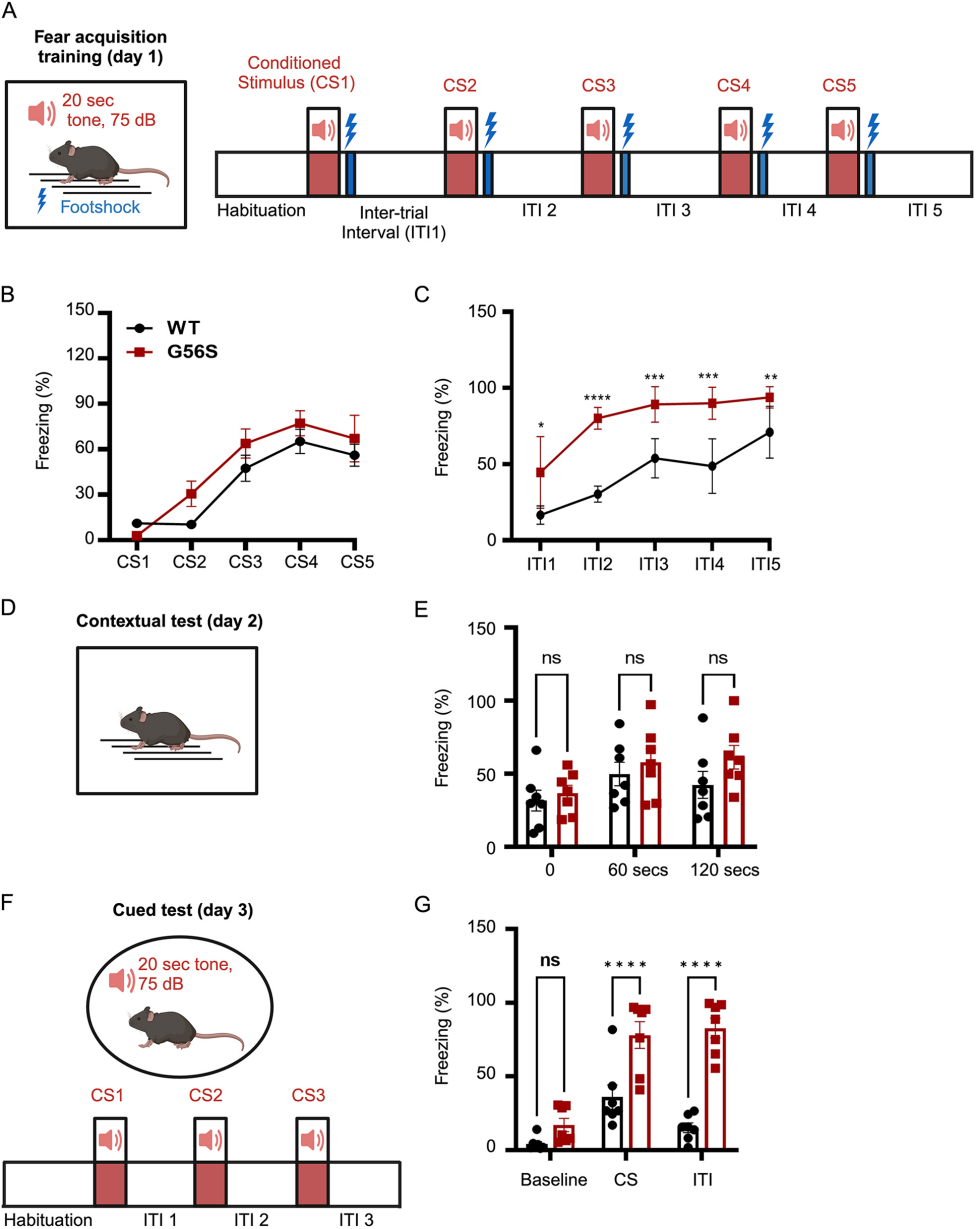
**Auditory-cued fear responses of 5-month-old G56S and wild-type mice.** (A) Illustration of fear acquisition training on day 1, in which the animals were subjected to sound stimulation for 20 s at 75 decibels, i.e. conditioned stimulation (CS), followed by foot shock and staggered inter-trial interval (ITI). (B,C) The fear response was expressed as the percentage of time spent in a stereotypical freezing state during CS (B) and ITI (C) periods. (D) Illustration of contextual test on day 2, in which the animal was placed in the same environment (indicated by a square cage), but without sound and shock stimulation. (E) The freezing state of the animals was recorded at the indicated times. (F) Illustration of cued-fear response on day 3, in which the animals were placed in a new environment (indicated by a circular cage) and provided with CS, i.e. sound stimulation for 20 s at 75 decibels, three times with no foot shock and variable ITIs. (G) The freezing state of the animals was recorded at baseline (during habituation), during CS and during ITIs. Data represent mean±s.e.m. from *n*=7 animals per group. *P<0.05; **P<0.01; ***P<0.001; ****P<0.0001 (two-way ANOVA analysis for repeated measures for B,C,E and unpaired two-tailed *t*-tests for G). ns, not significant. WT, wild type.

To complement the assessment of spatial learning through MWM, we measured stress-induced freezing using the fear conditioning test, which centers on complete tonic immobilization behavior due to innate, anti-predator, fear-related responses in rodents. The assay comprises three parts, namely fear acquisition training (Fig. 5A), followed by contextual (Fig. 5D) and cued (Fig. 5F) tests.

First, the mice were trained to associate sound (conditioned stimulus, CS) with a foot shock, with dedicated soundless intervals (inter-trial interval, ITI) between each stimulus. The stress-induced freezing time was recorded during the CS and the ITI (Fig. 5A). We observed a consistent increase in the freezing responses following the sound stimulation in both wild-type and G56S mice, which eventually plateaued (Fig. 5B), indicating similar rates of fear acquisition in both groups. However, during the ITIs, significantly longer and more frequent freezing responses were observed in the G56S mice (Fig. 5C), indicating more profound fear responses following stimulations than their wild-type counterparts.

On the second day, the mice were placed in the same experimental chamber, i.e. contextually similar, without any sound or electrical stimulation (Fig. 5D). We observed an increase in freezing response in the G56S mice, although it did not reach any statistical significance (Fig. 5E). On the final day, the mice were placed in a different test chamber for an initial 60 s habituation period, and subsequently provided with the sound stimulation, i.e. cued (Fig. 5F). The mutant mice showed clear elevated freezing percentages throughout the cued test compared with their wild-type counterparts (Fig. 5G). Taken together, these data suggest that the G56S mice exhibit heightened anxiety-related fear responses, which are typically present in neurological disorders.

### Reduced total and regional brain volumes in G56S mice

**Fig. 6.**
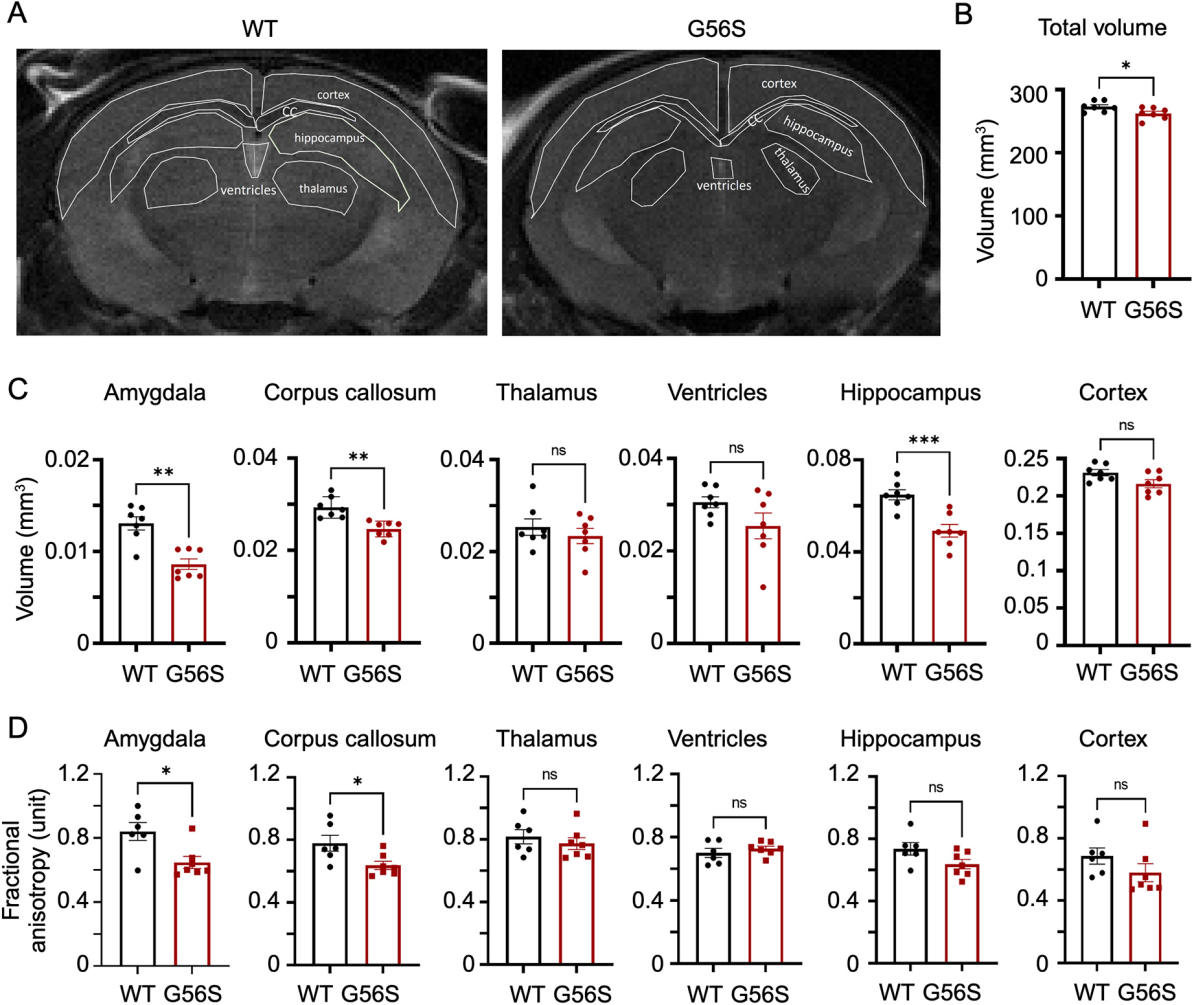
**Brain MRI of 18-week-old wild-type and G56S mice.** (A) Representative MRI images of coronal sections of wild-type and G56S brains. Annotations of different brain regions were based on the Allen Mouse Brain Atlas. (B,C) Volumetric analyses of the total brain volume (B) and volumes of the annotated regions (C) highlighted in A. Regional brain volumes were normalized to the total brain volumes. (D) Fractional anisotropy was quantified using DSI-Studio software. Comparisons of single variables between wild-type and G56S mice were performed using unpaired two-tailed *t*-tests. Data represent mean±s.e.m., *n*=5-7 mice per group. *P<0.05; **P<0.01; ***P<0.001. ns, not significant. WT, wild type.

Next, we determined whether neuroanatomical structures were impaired in the G56S mice. We assessed the brain volumes of 18-week-old G56S and wild-type mice using T2-weighted magnetic resonance imaging (MRI) (Fig. 6A) and found that the G56S mice had smaller total brain volumes (Fig. 6B). Furthermore, several regions, including the amygdala, corpus callosum and hippocampus, were also smaller in volume in the G56S mice than in their wild-type counterparts (Fig. 6C). Subsequently, a diffusion tensor imaging protocol was applied to interrogate the microstructural integrity of the brain. We observed a significant reduction in fractional anisotropy in the G56S amygdala and corpus callosum (Fig. 6D), which indicates disrupted fiber tracts in these regions. The amygdala is responsible for fear learning and emotional responses, whereas the hippocampus is involved in various cognitive functions. Collectively, the reduction in brain volume and disruption of microstructural integrity, particularly in the amygdala and hippocampus regions, largely support the behavioral findings seen in the G56S mice.

### SMS deficiency alters transcriptomic profiles in the G56S brain cortical region

To interrogate in an unbiased manner the molecular mechanisms underlying some of the observed phenotypic abnormalities, we performed transcriptomic analysis on RNA isolated from G56S and wild-type brain cortex. The G56S cortex exhibited a significant decrease in spermine content (Fig. S3), similar to the finding in total brain (Fig. 1G). We focused on the cortex because of its role in directing higher complex tasks, including learning, memory and consciousness. Furthermore, previous studies suggest that spermine may have a protective role within the cerebral cortex (Kesler et al., 2009; Clarkson et al., 2004).

**Fig. 7.**
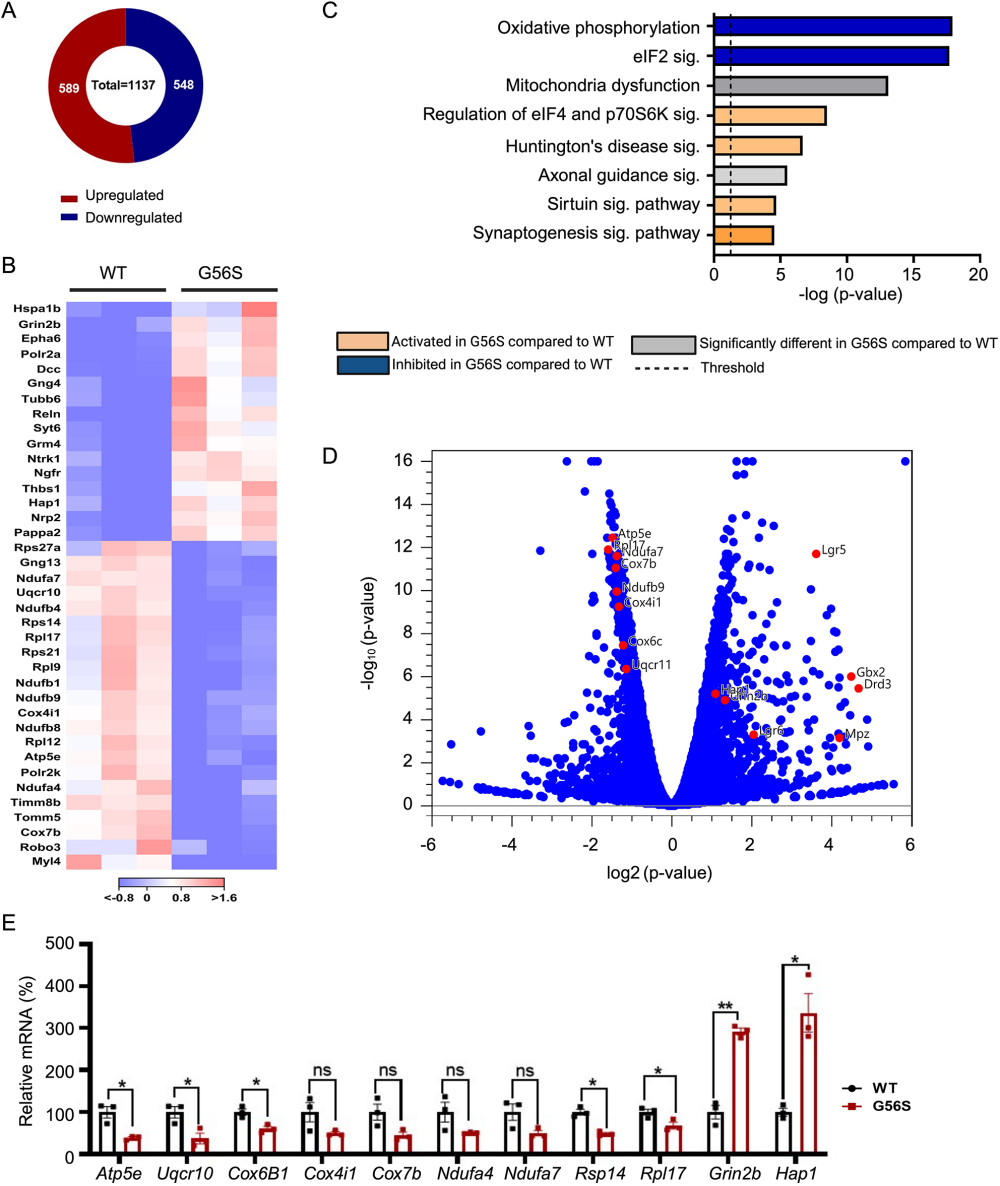
**Transcriptomic analysis of brain cortex from 18-week-old wild-type and G56S mice.** (A) Comparison of brain cortical region between wild-type and G56S mice revealed 1137 differentially expressed genes (DEGs), comprising 589 upregulated and 548 downregulated transcripts. (B) Heatmap of selected genes that exhibit statistically significant differences in expression (*P*<0.05) and absolute values of log_2_-fold change (LFC) greater than or equal to 1. (C) Different biological pathways identified by gene enrichment analysis of the upregulated and downregulated transcripts with *P<*0.05 and absolute LFC≥0.5. (D) Volcano plot showing the relative expression of selected genes involved in oxidative phosphorylation. (E) qPCR validation of selected genes implicated in oxidative phosphorylation, eukaryotic initiation factor 2 (eIF2) signaling and Huntington's disease. Data represent mean±s.e.m. from *n*=3 animals per group. *P<0.05; **P<0.01 (unpaired two-tailed *t*-tests). ns, not significant. WT, wild type.

Our data revealed more than 1000 differentially expressed genes (DEGs) between 18-week-old wild-type and G56S mice (Fig. 7A, Table S2), for which, after statistical filtering, the top 38 DEGs are presented as a heatmap (Fig. 7B). Gene enrichment pathway analysis revealed inhibition of pathways involved in mitochondrial oxidative phosphorylation (OXPHOS) and eukaryotic initiation factor 2 (eIF2) signaling crucial for ribosome protein synthesis, as well as activation of Huntington's disease, sirtuin and synaptogenesis signaling pathways (Fig. 7C). Some of these genes were further visualized on Volcano plot (Fig. 7D) and confirmed by qRT-PCR (Fig. 7E). Specifically, we observed decreased expression of several genes involved in OXPHOS, such as *Atp5e*, *Uqcr10* and *Cox6B1*. Of note, the expression of other OXPHOS-related genes, such as *Cox4i1*, *Cox7b*, *Ndufa4* and *Ndufa7*, were also reduced, although they did not reach statistical significance. Furthermore, there were decreases in the expression of *Rpl17* and *Rsp14* (both implicated in ribosome protein synthesis via eIF2 signaling), as well as increases in the expression of *Hap1* (Huntington-associated protein 1) and *Grin2b* (ionotropic NMDA receptor subunit 2b). Collectively, the transcriptomic data presented here outline several cellular processes, including but not limited to mitochondrial OXPHOS, that transpire from SMS deficiency and altered polyamine content in the brain.

### SMS deficiency impairs mitochondrial respiration in murine hippocampal cells

**Fig. 8.**
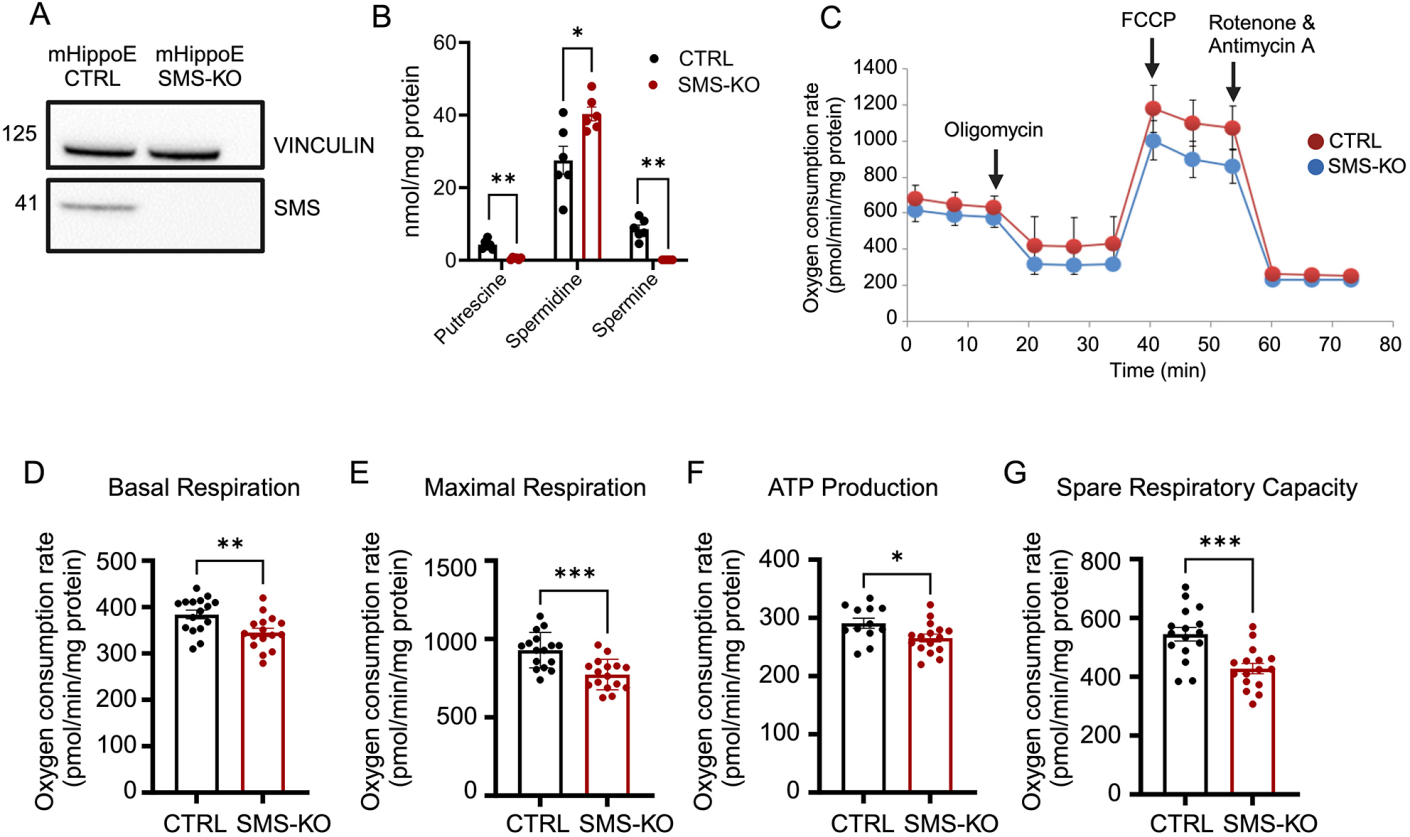
**Mitochondrial respiration in *Sms* knockout murine hippocampal cells.** (A,B) An SMS-deficient *in vitro* model (SMS-KO) was generated using CRISPR-mediated deletion in murine embryonic hippocampal (mHippoE) cells. SMS protein expression in the SMS-KO and control (CTRL) mHippoE cells was assessed by western blotting (A) and polyamine content measured by HPLC (B). (C) Mitochondrial respiration profiles of CTRL (red line) and SMS-KO (blue) cells. Oligomycin (ATP synthase inhibitor), FCCP (H^+^ ionophore) and rotenone/antimycin (mitochondria complex I/III inhibitors) were added at the indicated times. (D-G) Comparison of basal respiration (D), maximal respiration (E), ATP production (F) and spare respiratory capacity (G) between mHippoE SMS-KO and CTRL cells assessed using a Seahorse XFe96 analyzer. Data represent mean±s.e.m. from *n*=16 technical replicates of three independent experiments. *P<0.05; **P<0.01; ***P<0.001 (unpaired two-tailed *t*-tests).

Finally, we sought to perform functional validation on the impact of SMS deficiency on mitochondrial OXPHOS. We deleted the *Sms* gene in mouse embryonic hippocampal cells (mHippoE-14) using CRISPR-mediated knockout (SMS-KO) (Fig. 8A), resulting in altered polyamine content (Fig. 8B). We subsequently assessed the mitochondrial respiration using a Seahorse Bioanalyzer (Fig. 8C) and found a significant reduction in basal respiration, maximal respiration, rate of ATP production and spare respiratory capacity (Fig. 8D-G) in the SMS-KO cells, compared with control cells. In parallel, we isolated primary fibroblasts from G56S and wild-type mice (Fig. S4) and measured their mitochondrial respiration. Similar to the SMS-KO hippocampal cells, G56S fibroblasts also exhibited a significant reduction in basal and maximal respiration, ATP production and spare respiratory capacity. Taken together, these data strongly suggest that SMS deficiency and impaired polyamine metabolism alter mitochondrial bioenergetics and functions, which may contribute to the disease pathogenesis.

## DISCUSSION

In this study, we present a detailed characterization of the G56S mouse model carrying a missense *Sms* mutation, which recapitulates variants in patients with severe forms of SRS. We first demonstrated that the G56S mice lack SMS protein, resulting in high tissue spermidine levels and spermidine/spermine ratio. Furthermore, we showed that the G56S mice have small stature, with evident failure to thrive and reduced fat content, yet slightly increased lean muscle mass. Increased spermidine has been implicated in promoting lipolysis (Monelli et al., 2022), which may largely explain the reduction in body fat in the G56S mice. It is also possible that the absence of SMS or alteration in the spermidine/spermine ratio impairs mitochondrial functions (Ramsay et al., 2019; Li et al., 2017). In this case, the mice will depend more on glycolysis as a means of energy generation, thereby resulting in increased energy expenditure and less body fat. The decreased body weight and short stature seen in the G56S mice are consistent with the notion that disturbances in polyamine homeostasis impair cell growth and tissue development (Kemaladewi et al., 2018), which may lead to general growth failure.

We also observed low bone mineral density in the G56S mice. Although most SRS-affected individuals, including those with the G56S variant (de Alencastro et al., 2008), are eventually diagnosed with kyphoscoliosis, no scoliosis was detected in micro-CT scans of these mice. However, we cannot rule out the possibility that abnormal spines may develop in older mice.

The G56S mice display signs of cognitive impairment, reduction in exploratory behavior and heightened fear responses, which strongly indicate the existence of neurological abnormalities similar to those reported in many SRS-affected individuals. Given that polyamines are involved in the development of the nervous system (Slotkin and Bartolome, 1986), specific brain regions might be contributing to these behavioral defects. Indeed, the volumes of the amygdala and hippocampus, which are involved in fear-associated memory and learning, are decreased in the G56S mice, similar to reported observations in humans (Kesler et al., 2009). The MRI analysis also suggests brain atrophy, as indicated by a decrease in the fractional anisotropy value. Thus, these results indicate that impaired polyamine metabolism and excess spermidine accumulation might cause atrophy and neuronal loss in these regions (Kesler et al., 2009), which could manifest as impaired behavioral and learning outcomes.

Disruption of the polyamine pathway in the central nervous system has previously been associated with abnormal behavioral defects in the Dach-SMOX mouse model, which exhibits overexpression of spermine oxidase and overactive spermine catabolism (Cervetto et al., 2016; Leonetti et al., 2020). Consequently, the Dach-SMOX mice have decreased spermine and elevated spermidine and by-products of spermine catabolism in the cerebral cortex (Cervetto et al., 2016; Leonetti et al., 2020). Importantly, these mice show greater susceptibility to epileptic seizures and are thus used as a tool to evaluate treatment for epilepsy (Leonetti et al., 2020; Marcoli et al., 2022). Given that epileptic seizure is one of the significant clinical presentations in patients with SRS (Schwartz et al., 2013; de Alencastro et al., 2008; Larcher et al., 2020), it will be crucial to interrogate whether SMS deficiency manifests as epileptic seizure in the G56S mice. Overall, disruption of the polyamine pathway in both the Dach-SMOX and the G56S mice has significant consequences on central nervous system pathophysiology.

One potential mechanism that explains the behavioral defects observed in G56S mice is the spermidine-mediated disruption of receptor signaling. In an earlier study, Rubin et al. (2004) reported that intra-amygdala administration of spermidine in an experimental rat model resulted in a dose-dependent increase in freezing responses. These results suggested that the excess accumulation of spermidine in the brains of G56S mice might contribute to the observed increase in anxiety-related behaviors. However, the precise mechanisms underlying spermidine-mediated increases in fear responses remain unknown.

Spermidine may regulate the function of the amygdala via interactions with and modulation of the ion channel receptor for N-methyl-D-aspartate (NMDA). An earlier report detailed polyamine-mediated negative regulation of this receptor (Subramaniam et al., 1994). Administration of arcaine, a putative competitive antagonist at the polyamine binding site of the NMDA receptor, decreased spermidine-induced fear responses in rats (Rubin et al., 2004). Collectively, these results suggest that spermidine levels may impact amygdala function and that excess accumulation of spermidine may induce a fear response and other behavioral abnormalities seen in the G56S mice.

In addition to the neuroanatomic defects, the transcriptomic analysis revealed other potential mechanisms contributing to the phenotypic abnormalities observed in the G56S mice. These include impaired mitochondrial function, alterations in ribosomal protein synthesis signaling pathways and upregulation of genes implicated in the pathogenesis of Huntington's disease. Although not elucidated further in our study, mitochondrial dysfunction has been implicated in various neurological or neurodegenerative diseases (Guo et al., 2013), including in SRS (Ramsay et al., 2019; Li et al., 2017). Decreased mitochondrial respiration in isolated fibroblasts from patients with SRS was also observed in an independent study (C. Schwartz, personal communication). Increased spermidine levels that accumulate in cells that lack SMS may promote the synthesis and release of reactive oxygen species (ROS), which induce mitochondrial oxidative stress and impaired mitochondrial function (Li et al., 2017). Furthermore, earlier reports suggest that spermine modulates mammalian mitochondrial translation initiation processes (Lee et al., 2021; Christian et al., 2010). Thus, the lack of SMS and spermine in the G56S mice may inhibit the synthesis of mitochondrial proteins and potentially result in impaired mitochondrial functions. In addition, normal mitochondrial metabolism can result in the accumulation of potentially damaging levels of by-products, including ROS and Ca^2+^ (Zorov et al., 2014). As a polycationic molecule, spermine is known to have the potential to scavenge mitochondrial ROS (Ha et al., 1998; Sava et al., 2006) and reduce the levels of mitochondrial permeability transition pore (mPTP) generated in response to Ca^2+^ accumulation (Elustondo et al., 2015). Thus, the lack of SMS or an observed decrease in cellular spermine content may result in mitochondrial damage. Finally, it is also possible that mitochondrial impairment in SRS may relate to the decreased expression of nuclear genes encoding mitochondrial proteins reported in this study. Although we do not yet understand how *Sms* mutations and/or the decrease in spermine content result in the changes in gene expression pattern observed in this study, either factor may be involved in direct or indirect interactions with crucial transcription factors. Identifying these relevant transcription factors will be important to improve our understanding of how spermine and/or SMS modulate mitochondrial functions.

Finally, we acknowledge that natural history studies in mice are a valuable way to understand disease progression. Indeed, longitudinal assessments of mobility and anxiety in the open-field assay show developmental changes over time (Fig. 3), indicating phenotypic deterioration of the G56S mice. However, the remainder of the behavioral assays and neuroanatomical assessments were performed at a single time point, which prevented us from capturing the age-related decline in a comprehensive manner. Such limitation was largely attributed to the difficulty in obtaining a sufficient number of hemizygous G56S mice. As shown in Fig. S5, the rate of obtaining male hemizygous G56S mice was below the typical Mendelian ratio. This is likely due to embryonic lethality; however, interrogating such a mechanism is out of the scope of this study. These observations were not unique to our facility and have been reported by other laboratories (O. Phanstiel IV, personal communication, Snyder-Robinson Foundation Conference). During our disease characterization study, an effort has been made to swap the genetic backgrounds from C57BL/6J (described in this article) to B6C3H, which is a mix of C57BL/6J and C3H/HeJ backgrounds, to improve the breeding quality. This new strain B6C3H-Sms^em2Lutzy^/J, (The Jackson Laboratory, #033707), is commercially available and can be incorporated for future pre-clinical assessment of therapeutic interventions for SRS.

In conclusion, efforts to develop effective therapies for SRS will require a better understanding of the disease pathophysiology as well as suitable mutation/variant-specific animal models that recapitulate many of the clinical manifestations in the affected individuals. The findings presented in this study suggest that the G56S mouse is a good model that can be used to study SRS pathogenesis and serve as an important tool for therapeutic development. Several therapeutic interventions that are currently in development focus on rebalancing the spermidine/spermine ratio using polyamine analogs (Stewart et al., 2023; Tantak et al., 2021) or difluoromethylornithine to slow down putrescine production (Stewart et al., 2023), or alleviating the effect of spermidine-induced ROS using antioxidants (Li et al., 2017; Tao et al., 2022). Gene therapy and genome editing are gaining momentum in the rare disease space (Papaioannou et al., 2023) for which mutation in *SMS* in SRS would be a suitable target. Our study lays a crucial foundation and provides useful parameters that may be adopted in efforts to assess the efficacy of any therapeutic agents and/or improve current clinical management of SRS.

## MATERIALS AND METHODS

### Mice

All animals used in this study were housed at the University of Pittsburgh Division of Laboratory Animal Resources, Rangos Research Building, which was approved by the University of Pittsburgh's Institutional Animal Care and Use Committee (protocol number 2206137). The colony of mutant mice was established by breeding female heterozygous *Sms* mutation carriers (C57BL/6J-Sms^em2Lutzy^/J; The Jackson Laboratory, 031170) and male wild-type C57BL/6J mice (The Jackson Laboratory, 000664). The male offspring of this cross that harbored the X-linked G56S *Sms* mutation and wild-type littermate controls were used in the experiments described in this study. To ensure that only male mice harboring the desired mutation were used, pups were genotyped at Transnetyx using the following probes: forward primer, 5ʹ-ACCTGGCAGGACCATGGATATTTA-3ʹ; reverse primer, 5ʹ-GTGTTCACATCTAAAGCCCATGAGA-3ʹ; reporter 1, 5ʹ-AACAAGAATGGCAGGTAAG-3ʹ; and reporter 2, 5ʹ-ACGAACAAGAATTCCAGG-3ʹ.

### Open-field activity assay

The open-field chamber is a hollow square field box equipped with tracking software (ACTITRACK, Panlab/Harvard Apparatus) connected to an infrared tracking system that monitors animal movement. The walls of the box were opacified (covered with aluminum foil) to prevent the environment from influencing the behavior of the mouse undergoing testing. The chamber was divided into two imaginary zones: an outer zone (45×45 cm) and an inner or center zone (18.5×18.5 cm, centered at 22.5 cm from the wall on each side). Experiments were undertaken under constant room temperature (22-25°C) and light levels. The mice were habituated in the procedure room for 15 min each time before the assay was initiated. This was done to reduce any stress on the mice before the tests were conducted. Each mouse was released at the same location near the wall of the box and movement was evaluated for 15 min using the infrared tracking system. The positions recorded for each mouse were used to generate tracking plots and to determine the distance traveled, speed and time spent in each zone (i.e. within the entire apparatus and specifically in the center zone). The total amount of time spent and the type of body motion (i.e. rearing, leaning and vertical activity) detected in the center zone were used as relative measurements of explorative behavior and anxiety-related responses, respectively.

### Auditory-cued fear conditioning

The conditioning procedure was carried out using a specifically designed chamber (model H10-11M-TC-SF, Coulbourn Instruments). The conditioning chamber (25×25×25 cm) had three gray methacrylate walls, a grid floor connected to a shock scrambler to deliver foot shock as the unconditioned stimulus (US), and a speaker mounted on the chamber ceiling to deliver audible tones as the conditioned stimulus (CS). The conditioning chamber was fitted with a high-sensitivity camera system that monitored animal movement. The chamber was confined in a ventilated, soundproof enclosure (78×53×50 cm) on an anti-vibration table in a quiet room. The door to the room remained closed throughout the conditioning and testing periods.

On the first day (fear acquisition), the animals were habituated for 120 s in the chamber before the delivery of CS-US pairs [i.e. a 75 dB tone (CS) for 20 s followed by a 15-sec trace and then foot shocks (US) of 0.6 mA for 2 s] with variable and pseudo-randomly distributed intervals between pairs of stimuli (90-203 s). On the second day (fear retention), the session started with the mice placed in the same environment. During this phase, the mice were provided with no stimulation that might elicit contextual fear responses. Freezing responses in this otherwise familiar environment were monitored.

For the third session, the mice were placed in a different environmental setting (i.e. a chamber with a covered floor and white walls) to assess the retention of cued fear in a novel context. Baseline fear responses were monitored for 90 s followed by the delivery of three CS (75 dB and 20 s) separated by variable inter-trial intervals (ITIs). The movement of the animal was sampled at a frequency of 50 Hz for quantitative analysis (Freezeframe, Coulbourn Instruments). Freezing behavior was analyzed during the delivery of the CS (20 s periods) as well as during the 15 s trace period that would ordinarily precede the US (not delivered) to monitor the associative fear response. Each animal was assessed individually throughout the tests. The animals were gently handled before, during and after the test to avoid introducing any additional potential stress before or during each test that could influence the measured responses.

### MWM task

The MWM task was performed in a circular pool containing water using the procedure described by Tsien et al. (1996) with slight modifications. The animals were trained to find an escape platform that was submerged in the water. The training protocol (hidden platform, used to evaluate spatial learning) included five sessions with four trials per session per day. Navigation was tracked by a video camera and the escape latency (i.e. the time required to locate the platform) was recorded. An animal that failed to locate the platform within 90 s was guided to the platform. We then performed visible (to measure spatial memory) and probe (to measure non-spatial memory) tests on day six. In the visible test, colored tape was placed at the top of the platform. For the probe test, the platform was removed; the mice were allowed to swim in the pool for 60 s, and the time spent in each quadrant of the pool was recorded. The visual acuity of the mice in the pool was confirmed by changes in the swimming direction when approached by the technician carrying out the test. The acquired data was analyzed using the ANY-maze software.

### *In vivo* MRI scans

All mice were subjected to *in vivo* brain imaging while under isoflurane anesthesia. The mice were placed in a clear plexiglass anesthesia induction box that permitted unimpeded visual monitoring. Induction was achieved by the administration of 3% isoflurane in oxygen for several minutes. The depth of anesthesia was monitored by the toe reflex (extension of limbs, spine positioning) and respiration rate. Once established, the appropriate level of anesthesia was maintained by continuous administration of 1-2% isoflurane in oxygen via a nose cone. The mice were then transferred to the designated animal bed for imaging. Respiration was monitored using a pneumatic sensor placed between the animal bed and the mouse's abdomen. Rectal temperature was measured with a fiber optic sensor and maintained with a feedback-controlled source of warm air (SA Instruments).

*In vivo* brain MRI was carried out on a Bruker BioSpec 70/30 USR spectrometer (Bruker BioSpin MRI) operating at 7-Tesla field strength and equipped with an actively shielded gradient system and a quadrature radio-frequency volume coil with an inner diameter of 35 mm. Multi-planar T_2_-weighted anatomical images were acquired with a Rapid Imaging with Refocused Echoes (RARE) pulse sequence with the following parameters: field of view (FOV)=2 cm, matrix=256×256, slice thickness=1 mm, in-plane resolution=78×78 µm, echo time (TE)=12 msec, RARE factor=8, effective echo time (ETE)=48 ms, repetition time (TR)=1800 ms, and flip angle=180°. Multi-planar diffusion MRI was performed using the following parameters: FOV=2.0 cm, matrix=128×128, slice thickness=1.5 mm, in-plane resolution=156×156 µm, TE=16.31 ms, TR=1500 ms, diffusion preparation with the spin echo sequence, diffusion gradient duration=4 ms, diffusion gradient separation=8 ms, diffusion direction=30, number of A_0_ images=1, and b value=1500 s/mm^2^.

The MRI data were exported to a DICOM format and analyzed using the open-source ITK-SNAP (http://www.itksnap.org) brain segmentation software by two independent observers who were unaware of the experimental conditions. The volumes of each region of interest (ROI), including the amygdala, corpus callosum, thalamus, ventricles, hippocampus and cortex, were manually drawn by observers unaware of experimental groupings based on the information obtained from the Allen mouse brain atlas (https://mouse.brain-map.org/static/atlas). To account for potential differences in the sizes of brains in G56S and wild-type mice, volumes from each brain region were normalized to the total brain volume of each mouse.

Diffusion MRI was analyzed using the open-source DSI studio (http://dsi-studio.labsolver.org/) to obtain fractional anisotropy (FA). ROIs contributing to quantitative and statistical analyses, including the cortex, hippocampus, thalamus, corpus callosum and ventricles with cerebrospinal fluid (CSF), were manually segmented and defined by independent observers unaware of experimental groupings.

### *In vivo* micro-CT scans

All mice undergoing *in vivo* micro-CT imaging were maintained under general inhalation anesthesia with isoflurane as described for MRI scans above. Once established, anesthesia was maintained with 1.5% isoflurane in oxygen administered using a nose cone, and the mouse was transferred to the designated animal bed for imaging. Respiration was monitored as described above. Respiration gating was performed using a BioVet system that was triggered by maximal inhalation with a 500 ms trigger delay.

Respiration-gated *in vivo* micro-CT imaging was performed with Siemens Inveon Multimodality micro-CT-SPECT-PET system with the following parameters: full rotation, 360° projections; settle time 1000 ms; 4×4 binning; effective pixel size of 76.75 µm; trans axial FOV 78.6 mm with 4096 pixels; axial FOV 76.1 mm with 3968 pixels 80 kV of voltage; current of 500 µA; exposure time of 410 ms. The 3D micro-CT images were reconstructed using the Feldkamp algorithm and were calibrated in Hounsfield units (HU). Double distilled water was set at a readout of 0 and air at −1000 HU.

The 3D micro-CT image stacks were analyzed using the Inveon Research Workplace (IRW). The ROI analysis function was used with a thresholding tool that created several ROIs with different HU. A cylindrical 3D ROI was drawn around the body that encompassed the entire body. All external air around the mouse was excluded from the ROI and a custom threshold was set between 400 and 5700 HU to capture the bones. The mean HU values obtained from each ROI were used to quantify bone density.

### Body composition measurements

The body composition (percentage lean and fat weight) of the mice was measured by quantitative MRI (EchoMRI, Echo Medical Systems). Animals were placed in thin-walled plastic cylinders with plastic restraining inserts. Each animal was briefly subjected to a low-intensity electromagnetic field that measured total body composition. Percentages of fat and lean weights were determined based on total body weight.

### Cell lines

Primary fibroblast cells from the ears of the G56S and wild-type mice were isolated using the protocol described by Khan and Gasser (2016). The identity of the fibroblasts was confirmed by vimentin immunofluorescence staining against non-fibroblast sources. mHippoE-14 cells were purchased from Cedarlane, Canada. Cedarlane Canada performed authentication of the mHippoE-14 prior to shipping. Antibiotic-free supernatant was collected from all cell lines and tested for *Mycoplasma* annually. Cell lines used in this study were confirmed to be *Mycoplasma* negative.

### RNA isolation and quantitative PCR

Total RNA was isolated from mouse tissues using the Nucleospin RNA Plus kit (Macherey-Nagel, 740984.50), following the manufacturer's instructions. cDNA synthesis was performed using the iScript Reverse Transcriptase Supermix kit (Bio-Rad, 1708841) according to the manufacturer's instructions. Quantitative PCR (qPCR) was performed using a 2X SYBR Green Fast qPCR Mix kit (ABclonal, RM21203) in a C1000 Touch Thermal Cycler (Bio-Rad). The primer sequences used to amplify target genes of interest are listed in Table S1. The expression of endogenous *Gapdh* was used as an internal control to measure the relative expression of genes of interest. The 2^ΔΔC^ method was used to assess relative fold change in gene expression in tissue samples from wild-type and G56S mice. Values are presented as the percentage change in fold expression.

### RNA-sequencing (RNA-seq) and pathway enrichment analysis

After completing the RNA extraction procedure described above, samples were submitted to the Health Sciences Genomic Core at the UPMC Children's Hospital of Pittsburgh. RNA quality was determined using the Agilent Bioanalyzer 5300 Fragment Analyzer (Agilent Technologies). cDNA libraries were prepared using the Illumina Stranded mRNA library preparation kit (Illumina). Sequencing was performed using the NextSeq 2000 platform with pair-end 58 bp reads. Analysis of sequence reads, including quality control, mapping and generation of tables of DEGs, heatmaps and volcano plots were performed using QIAGEN licensed CLC Genomic Workbench software versus 22.0.1. Pathway enrichment analysis of the DEGs was performed using QIAGEN-licensed Ingenuity Pathway Analysis (IPA) software. The gene expression profile identified by RNA-seq was validated by qPCR as described above.

### *In vitro* analysis of mitochondria respiration (Seahorse assay)

Oxygen consumption rates were determined using a Seahorse XFe96 Extracellular Flux Bioanalyzer (Agilent Technologies). Cells were plated in a 96-well assay plate at a density of 10,000 (for the mHippoE-14 cell line) or 40,000 cells/well (for primary fibroblasts) and cultured overnight. The following day, cells were equilibrated with Seahorse XF base medium (Agilent Technologies) supplemented with glucose, sodium pyruvate and L-glutamine at 37°C in a non-CO_2_ incubator for 1 h before assay measurement. Mitochondrial function was assessed by sequential addition of 1.5 μM oligomycin, 1 μM FCCP [carbonyl cyanide-4-(trifluoromethoxy) phenylhydrazone] and 0.5 μM rotenone/antimycin A by the Seahorse Bioanalyzer. Data were normalized to the total protein content of the cells.

### Protein isolation, quantification and western blotting

Total proteins were extracted from tissues isolated from G56S and wild-type mice tissues using RIPA homogenizing buffer (150 μl of 50 mM Tris HCl pH 7.4, 150 nM NaCl, 1 mM EDTA) followed by homogenization using a bullet blender. After homogenization, 150 µl of RIPA double-detergent buffer (2% deoxycholate, 2% NP-40, 2% Triton X-100 in RIPA homogenizing buffer) supplemented with protease inhibitor cocktail (Roche, A32953) was added to the tissue homogenate followed by incubation on a shaker for 1 h at 4°C. The tissue homogenate was then centrifuged at 11,000* **g*** for 10 min at 4°C. The resulting supernatant was used to quantify total protein using the Pierce BCA protein assay kit (Thermo Fisher Scientific, 23225) according to the manufacturer's protocol. Twenty micrograms of total protein were fractionated on 4-12% gradient gel (Thermo Fisher Scientific, NP0336BOX). After proteins had separated on the gel, they were transferred by electroblotting onto a polyvinylidene fluoride (PVDF) membrane and blocked with 5% non-fat milk in 1× TBS containing 0.1% Tween 20. The membrane was then incubated overnight with rabbit anti-spermine synthase [Abcam, ab156879 (EPR9252B), 1:1000] or rabbit anti-vinculin [Abcam, ab129002 (EPR8185), 1:10,000]. After incubation with the primary antibody, the membranes were washed and then incubated with the secondary antibody (goat anti-rabbit IgG–HRP conjugate, Bio-Rad, 1706515, 1:5000 and 1:10,000 for the spermine synthase and vinculin sections of the membrane, respectively) for 1 h at room temperature. Specific protein bands were detected using SuperSignal West Femto Maximum Sensitivity Substrate (Thermo Fisher Scientific, 34095). Bands corresponding to immunoreactive SMS and vinculin were identified and quantified using the ChemiDoc Imaging System (Bio-Rad).

### Polyamine measurement

The polyamine content in isolated tissues was measured by precolumn dansylation, a high-performance liquid chromatography (HPLC) method described by Kabra et al. (1986) using 1,7-diaminoheptane as the internal standard.

### Statistical analysis

Statistical analysis was performed using GraphPad Prism software version 9.0. Each variable was statistically compared between the wild-type and G56S mice using either unpaired two-tailed *t*-test or two-way ANOVA with repeated measures. A *P-*value less than 0.05 was considered statistically significant.

## Supplementary Material

10.1242/dmm.050639_sup1 Supplementary information

## First Person

This article has an associated First Person interview with the first author of the paper.
