# Supplementary material for: Impaired polyamine metabolism causes behavioral and neuroanatomical defects in a mouse model of Snyder–Robinson syndrome
Source: Dis Model Mech. 2024 May 9;17(6):dmm050639. doi: 10.1242/dmm.050639 (PMC11103582; doi:10.1242/dmm.050639)
Supplement: Supplementary information [file dmm-17-050639-s1.pdf]

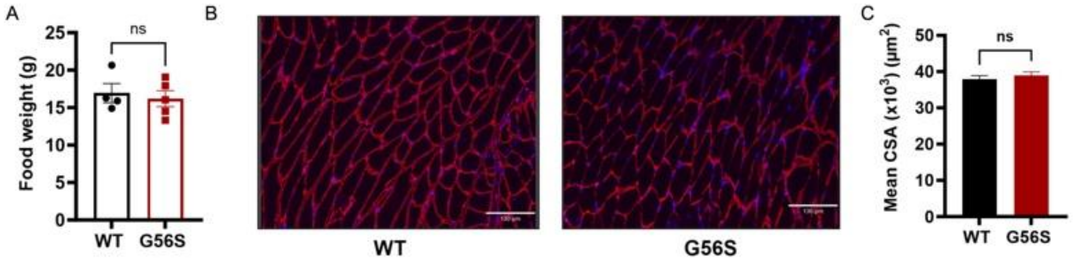

Supplementary Fig. 1

Fig. S1. Comparison of food consumption and muscle fiber size between wild-type and G56S mice. A. 5-day food consumption monitoring of G56S and age-matched WT mice using a comprehensive laboratory animal monitoring system (CLAMS). **B.** Immunofluorescence images of WT and G56S mice muscle fibers stained with anti-laminin antibody to detect laminin (red) and DAPI to detect the nuclei (blue). **C.** Quantification of muscle fibers cross-sectional area (CSA) using ImageJ software. Data represent mean ± S.E.M, n=3 mice per group. ns, not significant.

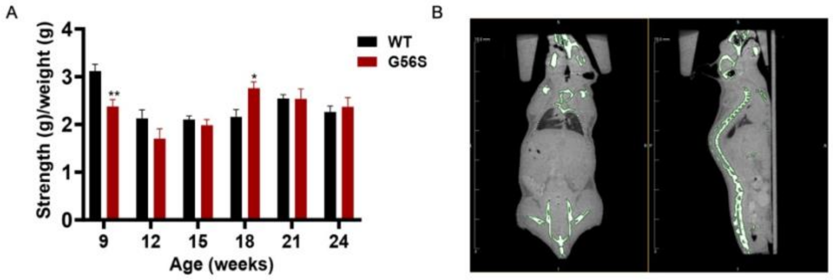

Supplementary Fig. 2

Fig. S2. Comparison of grip strength and bone density between wild-type and G56S mice. A. Forelimb grip strength of the G56S and WT mice, normalized to the body weight of the animals. Data is presented as mean ± S.E.M, n=3 mice per group. *$p < 0.05$; **$p < 0.01$. **B.** Representative 3D micro-CT scan images of a mouse under anesthesia. Images were analyzed using the Inveon Research Workplace (IRW) and a threshold was applied to exclude soft tissues. The remaining dense tissue (bone, colored white) was subsequently quantified (green line lines around the dense bone images). Note: The scan captured about 90% of the animal's body without the tail.

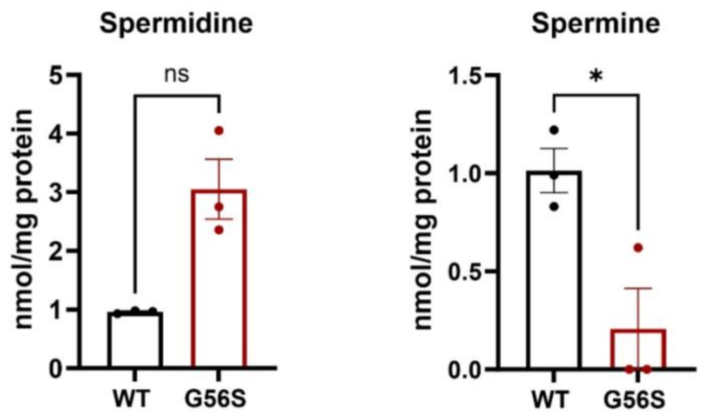

Supplementary Fig. 3

Fig. S3. The polyamine content of the brain cortex of 18-week-old wildtype and G56S mice as quantified by HPLC. The putrescine level was below the limit of detection. Data represent mean ± S.E.M from n = 3 mice per group; ns = not significant, *$p < 0.05$.

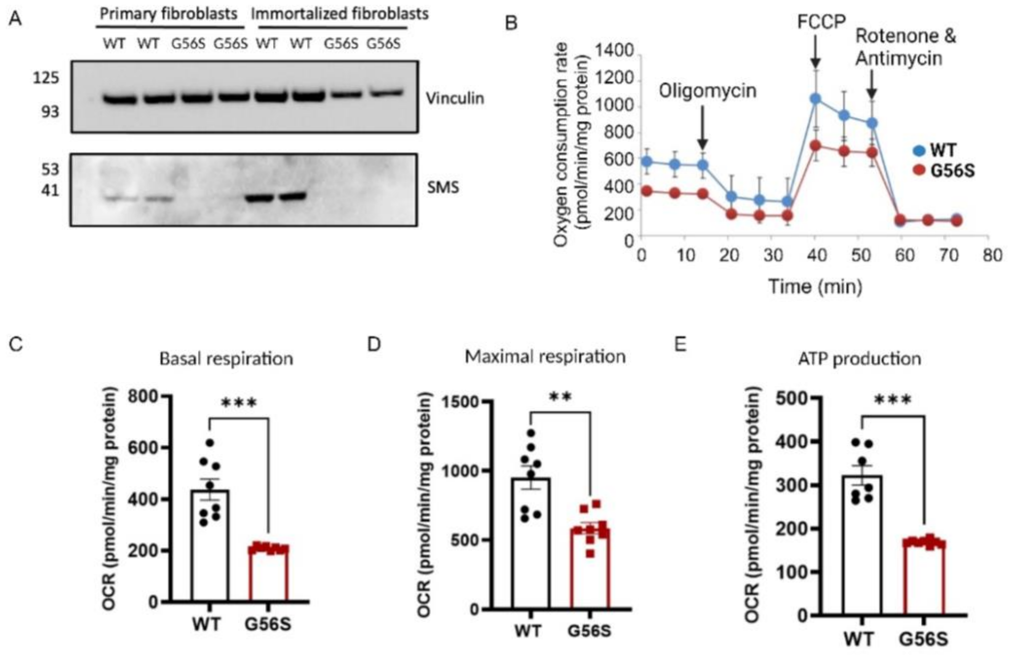

Supplementary Fig. 4

Fig. S4. Impaired mitochondrial bioenergetic in G56S-derived fibroblasts. A. SMS protein expression in primary fibroblasts isolated from ear clips of WT and G56S mice. Immortalized lines generated in parallel were also analyzed for SMS protein. **B.** Respiratory profiles of WT and G56S primary fibroblasts. Oligomycin (ATP synthase inhibitor), FCCP (H^+ ionophore), and rotenone/antimycin (mitochondria complex I/III inhibitors) were added at the times indicated. **C-E.** Basal respiration (**C**), maximal respiration (**D**), and ATP production (**E**) in WT and G56S primary fibroblasts were assessed using a Seahorse XFe96 analyzer. Data represent mean ± S.E.M. of n=8 technical replicates of two independent experiments. **$p < 0.01$; ***$p < 0.001$.

Disease Models & Mechanisms • Supplementary information

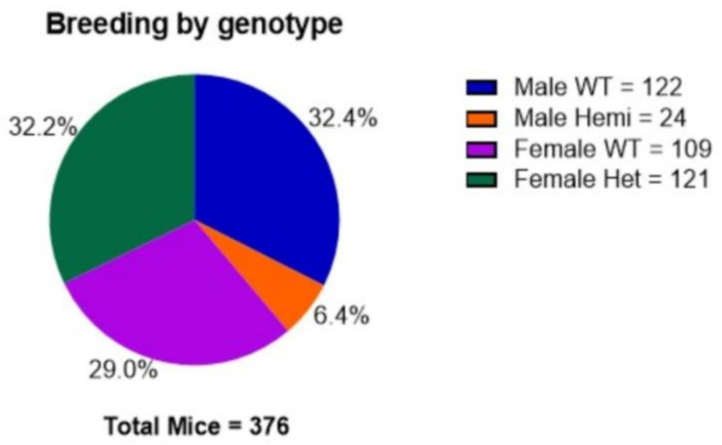

Supplementary Fig. 5

Fig. S5. Total number of WT, heterozygous, and hemizygous mice on the C57BL/6J background generated within one year. Breeding was set up by pairing female heterozygous mice with WT male mice.

Table S1. qPCR primer sequences

	Name	qPCR Primer Sequence (5' → 3')	Note
1	Mouse SMS	CCACACTATGGCAGCAGCAAG	Forward
		TGCACTGACTCTGTCATCCCC	Reverse
2	Mouse GAPDH	CTCCCACTCTTCCACCTTCG	Forward
		GCCTCTCTTGCTCAGTGTCC	Reverse
3	Mouse ATP5e	TACTCTGAAGCGACCCAGCG	Forward
		GCGTTCGCTTTGAACTCGGT	Reverse
4	Mouse Cox7B	TAGTCGCCGCAGTTCCATCT	Forward
		GCCACCACTTGCTGAATGCT	Reverse
5	Mouse Uqcr10	ACGCGATCTACGAGCACATCA	Forward
		GTCGGTGAACGGCAACTTGAAA	Reverse
6	Mouse RPS14	ATCAAACTCCGGGCCACAGG	Forward
		TGACATCCTCAATCCGCCCA	Reverse
7	Mouse RPL17	TTCCTGTAAGCGGCCAGAGG	Forward
		GCATTCCCTTGATGGCCTGG	Reverse
8	Mouse Hap1	GGCTGAGGAGCTCCGAACAT	Forward
		TCCCTGCAGTGAGTGTCACG	Reverse
9	Mouse GRIN2B	CGGAGCTGGCATCCGAATACA	Forward
		TGGAGCGTGGTCATTCCCAA	Reverse
10	Mouse Cox4i1	GTCTTGGTCTTCCGGTTGCG	Forward
		CATGTGCTCGAAGGCACACC	Reverse
11	Mouse Cox6B1	GAGTGGTACCGGCGTGTGTA	Forward
		TGCCTTCAGCTATGCGGTCA	Reverse
12	Mouse Ndufa4	GCAAGCCAAGAAGCATCCCA	Forward
		GTGCCAAGCGCATCACATACA	Reverse
13	mNdufa7	TCCTCGGGACAGAGTCGTCA	Forward
		CGCTTGGCGATCTCCTGGTA	Reverse

Disease Models & Mechanisms • Supplementary information

Table S2. List of differentially regulated genes between WT and G56S cortex

Downregulated genes								Upregulated genes						
GENE ID	Chr.	Max group mean	Log$_2$ fold Δ	Fold Δ	P-value	FDR p-value		GENE ID	Chr.	Max group mean	Log$_2$ fold Δ	Fold Δ	P-value	FDR p-value
Icam4	9	1.232	-1.081	-2.116	0.015256	0.049965978		Sall4	2	0.061	2.240	4.725	0.014972619	0.049296734
Itgae	11	0.086	-1.746	-3.355	0.0149964	0.04934519		Mki67	7	0.132	1.188	2.278	0.014911876	0.049128658
Gm29427	1	0.082	-6.163	-71.637	0.0147132	0.048544108		Hif3a	7	0.575	1.006	2.009	0.014636548	0.048336364
Gm15155	X	0.155	-2.533	-5.788	0.0142146	0.047145333		Serpine1	5	0.495	1.111	2.161	0.014609617	0.048263114
Zfp708	13	0.889	-1.018	-2.025	0.01367	0.045811738		Rtl9	X	0.414	1.036	2.051	0.014601504	0.048245012
Gm36028	16	0.129	-2.512	-5.704	0.0131286	0.044305362		Lbhd2	12	4.575	1.321	2.498	0.014141085	0.046952399
Cabp5	7	0.052	-2.680	-6.407	0.012376	0.042279546		Ccdc146	5	0.175	1.498	2.825	0.014018309	0.046671642
S100a8	3	1.455	-1.472	-2.774	0.0120425	0.041255871		C3	17	0.130	1.356	2.560	0.013858007	0.046255643
Cxcr2	1	0.045	-2.728	-6.626	0.0119643	0.041043567		Ptch2	4	0.255	1.221	2.331	0.013849803	0.046245114
Rps18-ps6	13	0.354	-2.608	-6.097	0.0117889	0.040594792		Dusp27	1	0.193	1.296	2.456	0.013096298	0.044225726
Nox1	X	0.035	-3.037	-8.208	0.0115929	0.040037389		Olfr550	7	0.065	2.041	4.115	0.01287429	0.043615912
Btn2a2	13	0.556	-1.247	-2.373	0.0115272	0.039870833		Zar1	5	0.464	1.727	3.310	0.012750287	0.043251807
2410137M14Rik	17	0.119	-3.658	-12.627	0.0114156	0.039559307		Tlr9	9	0.410	1.147	2.215	0.012681643	0.043082778
Prickle4	17	0.136	-6.374	-82.941	0.0112358	0.03912124		Padi1	4	0.032	3.656	12.603	0.012406248	0.042343515
Gm12166	11	0.423	-1.943	-3.845	0.011084	0.038666368		Insrr	3	0.107	1.485	2.800	0.01142127	0.039571557
Crybg2	4	0.202	-1.233	-2.351	0.0109537	0.038303114		Piezo2	18	0.119	1.224	2.337	0.011255273	0.039181698
Crybb3	5	0.948	-1.234	-2.353	0.0105557	0.037170073		Gas2l2	11	0.127	2.074	4.210	0.010224392	0.036219378
Gm49354	13	0.436	-2.504	-5.671	0.0103565	0.036595261		Lhcgr	17	0.105	1.439	2.712	0.010196621	0.03614197
Carlr	2	0.591	-1.057	-2.081	0.010068	0.035803999		Upb1	10	0.229	1.351	2.552	0.010155438	0.036031032
Aox2	1	0.036	-2.731	-6.641	0.0100032	0.035623463		Kcnj13	1	3.008	1.085	2.121	9.84E-03	0.035157132
Ugt2a2	5	0.112	-2.911	-7.523	9.21E-03	0.033177085		Prdm1	10	0.324	1.222	2.333	9.44E-03	0.033878918
Meltf	16	0.211	-1.289	-2.444	9.21E-03	0.033177085		Xkrx	X	0.481	1.209	2.311	9.14E-03	0.032988803
Gm49325	10	1.561	-1.891	-3.709	8.64E-03	0.031548506		Atp10b	11	0.248	1.016	2.023	9.10E-03	0.032871908
Mcmdc2	1	0.224	-1.164	-2.241	8.30E-03	0.030467473		Irs4	X	0.302	1.393	2.626	8.90E-03	0.032289358
Pla2g4b	2	0.080	-6.666	-101.533	8.16E-03	0.030048676		Slc16a8	15	0.198	3.201	9.197	8.85E-03	0.032171631
Gm27021	8	0.846	-1.480	-2.789	8.12E-03	0.029923006		Foxd2	4	0.246	1.542	2.912	8.80E-03	0.032000665
Ninj2	6	1.599	-1.122	-2.176	7.61E-03	0.028373499		Uncx	5	0.119	1.997	3.990	8.70E-03	0.031712493
Tmem37	1	1.299	-1.032	-2.044	7.52E-03	0.028081285		Ppl	16	0.263	1.253	2.383	8.58E-03	0.031363116
Cldn22	8	1.898	-1.375	-2.593	7.45E-03	0.027907129		Tjp3	10	0.216	1.448	2.729	8.56E-03	0.031292662
Theg	10	0.203	-1.941	-3.840	6.92E-03	0.026231111		Gm49387	14	0.170	6.812	112.398	8.45E-03	0.030950136
4930451I11Rik	7	0.450	-2.470	-5.542	6.83E-03	0.025906738		Pi16	17	0.233	1.403	2.645	8.35E-03	0.030596331
Gm14443	2	0.272	-1.367	-2.580	6.78E-03	0.025770946		Lyve1	7	0.404	1.348	2.546	8.21E-03	0.030205314
Il1b	2	0.385	-1.718	-3.291	6.33E-03	0.024308452		Sgcd	11	0.499	1.083	2.119	8.01E-03	0.029587199
Mslnl	17	0.110	-2.381	-5.209	6.29E-03	0.024218452		Rspo4	2	0.106	2.110	4.316	7.86E-03	0.029158838
Nxf7	X	0.530	-1.381	-2.604	6.26E-03	0.024107165		Art4	6	0.086	2.781	6.875	7.63E-03	0.028440374
Ms4a6b	19	0.697	-1.043	-2.061	5.96E-03	0.023145939		Casr	16	0.323	1.542	2.911	7.58E-03	0.028311324
Tsacc	3	2.120	-1.354	-2.557	5.94E-03	0.023082795		Drd2	9	6.856	1.004	2.006	7.26E-03	0.027284202
Cldn14	16	0.313	-1.664	-3.169	5.90E-03	0.022928132		Gabrr1	4	0.104	2.216	4.647	7.15E-03	0.026982211
Cpa2	6	1.275	-1.085	-2.121	5.74E-03	0.022418642		Col6a6	9	0.114	1.424	2.683	7.15E-03	0.026962991
Aldh3a1	11	0.754	-1.308	-2.476	5.65E-03	0.02212661		Cd109	9	0.306	1.124	2.180	7.08E-03	0.026729975
4930447C04Rik	12	0.224	-1.368	-2.581	5.39E-03	0.021313052		Prl	13	0.174	4.013	16.144	6.64E-03	0.025301073
Cklf	8	0.471	-1.061	-2.087	5.31E-03	0.021078633		Lbp	2	1.281	1.079	2.112	6.63E-03	0.025277187
Tph2	10	0.567	-1.231	-2.347	5.20E-03	0.020688469		Tspan10	11	0.113	4.278	19.396	6.30E-03	0.024224603
Riiad1	3	3.277	-1.010	-2.014	4.83E-03	0.019466421		Lpar3	3	0.241	1.573	2.976	6.25E-03	0.024107165
Dapk2	9	0.646	-1.162	-2.238	4.75E-03	0.019186352		Baiap2l1	5	0.398	1.407	2.651	6.12E-03	0.02369708
Espnl	1	0.140	-1.457	-2.746	4.67E-03	0.018906298		Omp	7	7.290	2.073	4.207	6.06E-03	0.023446773
Gm28040_1	1	0.507	-2.062	-4.176	4.58E-03	0.018628452		Hs3st3a1	11	0.700	1.159	2.233	5.97E-03	0.023167195
Hsbp1l1	18	0.996	-1.390	-2.621	4.36E-03	0.017855181		Xirp1	9	0.045	2.275	4.841	5.73E-03	0.022412724
Adam8	7	0.447	-1.294	-2.451	4.33E-03	0.017784998		Ripk4	16	0.204	1.490	2.810	5.69E-03	0.022278549
Tsks	7	0.356	-1.484	-2.797	4.33E-03	0.017761659		Otogl	10	0.134	1.386	2.614	5.60E-03	0.021988938
Cyp2a5	7	1.058	-1.812	-3.510	4.18E-03	0.017277402		Vmn1r206	13	0.090	2.165	4.483	5.37E-03	0.02126614
5430401F13Rik	6	0.442	-7.275	-154.900	4.12E-03	0.017088884		Trim58	11	0.070	4.007	16.082	5.33E-03	0.021139014
Gzma	13	0.254	-2.500	-5.656	4.11E-03	0.017036255		Ebf2	14	0.279	1.207	2.308	5.28E-03	0.020994739
Pde6g	11	0.302	-2.260	-4.789	4.10E-03	0.017031615		Krt73	15	1.003	1.051	2.073	5.27E-03	0.020926536
Arc	15	108.220	-1.098	-2.141	4.07E-03	0.016912551		Derpc	8	0.620	2.145	4.423	5.13E-03	0.020458671
Cyp11a1	9	0.969	-1.024	-2.034	3.87E-03	0.016198655		Ndor1_1	2	0.443	1.784	3.443	4.91E-03	0.019735353
Padi6	4	0.187	-1.888	-3.701	3.75E-03	0.01576052		Galr1	18	0.181	1.687	3.220	4.59E-03	0.018650884
Ankrd31	13	0.080	-1.721	-3.296	3.62E-03	0.01535137		Wdr72	9	0.039	4.383	20.864	4.53E-03	0.018444103
Cwh43	5	0.673	-1.186	-2.276	3.35E-03	0.014418289		Gm20517	17	0.078	7.181	145.156	4.45E-03	0.018153393
Shld3	13	1.470	-1.023	-2.033	3.26E-03	0.014111735		Ttc21a	9	0.353	1.378	2.600	4.43E-03	0.018104735
Ccdc18	5	0.335	-1.172	-2.253	3.21E-03	0.013926538		Rd3l	12	0.316	1.798	3.478	4.34E-03	0.017810698
Gm2004	2	0.255	-7.667	-203.241	3.19E-03	0.013868882		Gm21149	5	0.149	4.230	18.767	4.31E-03	0.017706449
Gm42420	6	1.160	-1.582	-2.993	3.15E-03	0.013731805		Sim1	10	0.044	2.159	4.467	4.30E-03	0.017684148
Gm49345	13	0.266	-2.785	-6.891	2.97E-03	0.013026242		Gsx2	5	0.318	1.828	3.549	4.20E-03	0.017351742
Myl2	5	0.332	-1.739	-3.338	2.75E-03	0.012189479		Krt5	15	0.119	2.788	6.908	4.17E-03	0.017243666

Fap	2	0.679	-1.083	-2.118	2.73E-03	0.01213112		Tram2	1	0.318	1.045	2.063	3.96E-03	0.016522911
Sftpc	14	2.187	-1.140	-2.203	2.70E-03	0.012039046		Cenpe	3	0.203	1.282	2.431	3.96E-03	0.016516251
Gm28040_2	1	0.717	-1.605	-3.042	2.68E-03	0.011959293		Nkx6-1	5	0.113	2.309	4.955	3.94E-03	0.016448186
Ptpn18	1	1.990	-1.033	-2.047	2.63E-03	0.011737559		Postn	3	0.766	1.055	2.078	3.91E-03	0.016328544
Il12a	3	1.087	-1.356	-2.559	2.27E-03	0.010443603		Fbxl7	15	0.404	1.222	2.332	3.79E-03	0.015926839
Sult1c1	17	0.235	-3.086	-8.493	2.26E-03	0.010394065		1700012B09Rik	9	0.942	1.633	3.101	3.57E-03	0.015181755
BC035044	6	1.279	-1.088	-2.126	2.20E-03	0.010186514		Krt2	15	1.196	1.102	2.146	3.57E-03	0.015181755
Ly6m	15	0.100	-3.208	-9.238	2.09E-03	9.76E-03		Mmp19	10	0.361	1.464	2.759	3.53E-03	0.015052811
Lypd2	15	1.000	-1.913	-3.766	2.09E-03	9.75E-03		Gm4767	10	0.147	1.797	3.475	3.33E-03	0.014341057
Hjv	3	0.584	-1.526	-2.880	2.04E-03	9.54E-03		Gxylt2	6	0.374	1.010	2.014	3.25E-03	0.014080706
Ccl6	11	1.782	-1.063	-2.089	1.91E-03	9.02E-03		Chrna10	7	0.469	2.280	4.856	3.24E-03	0.014038216
Zar1l	5	0.871	-1.622	-3.079	1.89E-03	8.96E-03		A730046J19Rik	X	0.157	1.845	3.593	3.23E-03	0.014009994
Fam174c	10	4.596	-1.072	-2.102	1.88E-03	8.91E-03		Avp	2	5.003	2.153	4.447	3.22E-03	0.0139575
Cd40	2	0.308	-1.644	-3.126	1.84E-03	8.79E-03		Ly75	2	0.243	1.202	2.301	3.14E-03	0.013680301
Rnf39	17	1.835	-1.004	-2.005	1.81E-03	8.66E-03		Zic3	X	0.757	1.010	2.014	3.10E-03	0.013517732
Gm28778	1	1.092	-2.205	-4.609	1.76E-03	8.47E-03		Nkx2-1	12	0.427	1.723	3.301	3.02E-03	0.01322799
Olfr464	11	0.400	-1.636	-3.107	1.76E-03	8.46E-03		Adamts5	16	0.348	1.005	2.006	2.78E-03	0.012312269
Gm3696	14	1.386	-1.048	-2.067	1.70E-03	8.20E-03		Fgf16	X	0.978	1.689	3.224	2.74E-03	0.012163341
Arr3	X	0.403	-1.726	-3.308	1.69E-03	8.18E-03		BC035947	1	0.488	1.388	2.618	2.64E-03	0.011780173
Hpx	7	0.561	-1.599	-3.028	1.47E-03	7.30E-03		Ak7	12	0.570	1.504	2.836	2.64E-03	0.011780173
Ocm	5	0.719	-1.896	-3.723	1.45E-03	7.22E-03		Gucy2f	X	0.203	1.291	2.447	2.37E-03	0.010785066
Gm6619	6	0.969	-3.401	-10.566	1.35E-03	6.76E-03		Nmb	7	10.408	1.474	2.778	2.29E-03	0.010488119
Golt1a	1	0.167	-2.676	-6.390	1.33E-03	6.72E-03		Slc38a8	8	0.200	2.283	4.867	2.24E-03	0.010346868
Ugt2a1	5	0.283	-3.508	-11.377	1.23E-03	6.26E-03		Cldn19	4	0.284	1.900	3.733	2.22E-03	0.010252521
Hes3	4	0.142	-2.864	-7.280	1.21E-03	6.20E-03		Flnc	6	0.348	1.107	2.153	2.21E-03	0.010215889
Ccnb1ip1	14	0.232	-2.225	-4.675	1.21E-03	6.20E-03		Fam167a	14	0.567	1.052	2.074	2.17E-03	0.010092077
Gm2296	9	0.432	-2.838	-7.149	1.21E-03	6.19E-03		Gm49368	7	0.052	3.419	10.695	2.09E-03	9.76E-03
Urah	7	0.750	-1.617	-3.068	1.21E-03	6.19E-03		Cdca7l	12	0.412	1.558	2.944	2.09E-03	9.76E-03
Spag6	2	1.015	-1.191	-2.284	1.20E-03	6.16E-03		Dsg2	18	0.366	1.094	2.134	2.02E-03	9.46E-03
Il11ra2	4	0.295	-2.116	-4.336	1.18E-03	6.05E-03		Hephl1	9	0.113	7.828	227.296	2.00E-03	9.39E-03
Spdya	17	0.655	-1.462	-2.754	1.17E-03	6.03E-03		Arhgef16	4	0.396	1.689	3.225	1.96E-03	9.21E-03
Olfr77	9	0.216	-1.259	-2.393	1.15E-03	5.95E-03		Lhx1	11	0.063	2.750	6.725	1.95E-03	9.19E-03
Vsig2	9	0.722	-1.609	-3.050	1.07E-03	5.60E-03		6430571L13Rik	9	3.415	1.111	2.160	1.94E-03	9.16E-03
Dazl	17	1.136	-1.000	-2.001	1.00E-03	5.31E-03		Slc43a3	2	0.504	1.811	3.508	1.90E-03	8.98E-03
Ccl9	11	0.985	-1.106	-2.153	8.05E-04	4.42E-03		Sfmbt2	2	0.315	1.098	2.140	1.86E-03	8.87E-03
Traf5	1	1.699	-1.023	-2.032	7.79E-04	4.31E-03		S100a5	3	18.688	2.913	7.531	1.75E-03	8.39E-03
Cenpa	5	1.425	-1.177	-2.260	7.55E-04	4.20E-03		Vash2	1	0.822	1.057	2.081	1.68E-03	8.12E-03
9430038I01Rik	7	0.611	-1.014	-2.020	6.76E-04	3.81E-03		Prss56	1	0.490	2.383	5.217	1.67E-03	8.07E-03
Slfn2	11	1.826	-1.122	-2.176	5.85E-04	3.40E-03		Col6a3	1	0.252	1.053	2.074	1.66E-03	8.04E-03
S100a4	3	4.855	-1.267	-2.406	5.65E-04	3.30E-03		Serpina9	12	1.264	1.427	2.688	1.58E-03	7.76E-03
Ptges3l	11	1.126	-1.231	-2.348	5.59E-04	3.27E-03		Prdm12	2	0.091	3.268	9.636	1.52E-03	7.51E-03
Tcap	11	4.094	-1.099	-2.142	5.31E-04	3.14E-03		Cubn	2	0.090	1.672	3.187	1.49E-03	7.37E-03
Gngt2	11	2.871	-1.058	-2.082	5.04E-04	3.00E-03		Emilin2	17	0.726	1.025	2.034	1.44E-03	7.14E-03
Fcor	8	11.272	-1.009	-2.012	4.38E-04	2.66E-03		Pcdhb21	18	0.659	1.039	2.055	1.43E-03	7.13E-03
Mpl	4	0.282	-2.027	-4.076	4.37E-04	2.66E-03		Pcdhb2	18	1.050	1.077	2.110	1.43E-03	7.11E-03
Gmfg	7	2.348	-1.419	-2.675	4.28E-04	2.61E-03		Fzd5	1	0.448	1.062	2.088	1.36E-03	6.84E-03
Prss22	17	1.166	-1.609	-3.051	3.97E-04	2.47E-03		Barhl2	5	0.700	1.676	3.195	1.32E-03	6.66E-03
Tmem232	17	0.918	-1.291	-2.447	3.60E-04	2.28E-03		Esyt3	9	0.689	1.317	2.491	1.23E-03	6.28E-03
Adamts13	2	0.462	-1.366	-2.578	3.40E-04	2.17E-03		Cdh23	10	0.212	1.201	2.299	1.12E-03	5.81E-03
Lat2	5	2.131	-1.220	-2.330	3.37E-04	2.16E-03		Spata18	5	0.308	2.117	4.338	1.10E-03	5.73E-03
Ly6d	15	0.643	-2.446	-5.447	3.29E-04	2.12E-03		Myh4	11	0.283	1.937	3.830	1.06E-03	5.55E-03
Kif4	X	0.617	-1.252	-2.382	3.27E-04	2.11E-03		Tmem26	10	0.092	2.539	5.813	1.05E-03	5.52E-03
Gm5617	9	7.211	-1.112	-2.162	3.07E-04	2.00E-03		Rab37	11	2.208	1.458	2.747	1.03E-03	5.42E-03
Cd164l2	4	2.925	-1.246	-2.371	2.95E-04	1.94E-03		Duox2	2	0.042	3.945	15.403	9.84E-04	5.23E-03
Fbxo17	7	1.793	-1.081	-2.115	2.95E-04	1.94E-03		Ghsr	3	0.097	2.449	5.462	9.05E-04	4.88E-03
Asb11	X	1.998	-1.121	-2.175	2.87E-04	1.90E-03		Col4a6	X	0.294	1.242	2.366	8.32E-04	4.55E-03
Rpe65	3	1.793	-1.313	-2.484	2.51E-04	1.69E-03		Alx3	3	0.995	1.385	2.612	8.14E-04	4.46E-03
B230307C23Rik	16	1.427	-1.102	-2.146	2.51E-04	1.69E-03		Mgam	6	0.072	8.357	327.981	7.93E-04	4.38E-03
Tmprss7	16	1.018	-1.252	-2.381	2.10E-04	1.46E-03		Gh	11	1.039	8.408	339.614	7.54E-04	4.20E-03
Npc1l1	11	0.243	-2.167	-4.492	2.07E-04	1.45E-03		Foxd3	4	0.387	8.417	341.707	7.45E-04	4.16E-03
Nanos3	8	0.763	-2.228	-4.684	2.04E-04	1.43E-03		Foxb1	9	0.096	3.231	9.392	7.30E-04	4.09E-03
Sap25	5	0.325	-5.508	-45.492	2.01E-04	1.41E-03		Tfap2c	2	0.248	2.523	5.747	7.21E-04	4.04E-03
Pagr1a	7	2.112	-2.461	-5.506	1.80E-04	1.29E-03		Lmod1	1	0.696	1.299	2.460	6.86E-04	3.86E-03
Gm11627	11	4.981	-1.085	-2.122	1.74E-04	1.25E-03		Gabre	X	0.234	1.701	3.252	6.71E-04	3.80E-03
Spata24	18	4.844	-1.060	-2.085	1.53E-04	1.12E-03		Trim67	8	0.719	1.043	2.060	6.42E-04	3.67E-03
Nudt6	3	2.450	-1.109	-2.157	1.53E-04	1.12E-03		Gm49496	13	0.845	8.535	370.982	6.26E-04	3.59E-03
Ctsk	3	2.757	-1.007	-2.009	1.44E-04	1.06E-03		Zim1	7	0.550	1.058	2.082	6.22E-04	3.58E-03
Cd7	11	1.580	-1.796	-3.473	1.25E-04	9.40E-04		Fign	2	0.362	1.103	2.147	6.14E-04	3.53E-03
Higd1b	11	4.627	-1.165	-2.243	1.08E-04	8.34E-04		Dlx6	6	4.211	1.012	2.017	5.66E-04	3.30E-03
Barx2	9	3.093	-1.042	-2.059	1.03E-04	8.04E-04		Siglec1	2	0.068	2.373	5.180	5.64E-04	3.30E-03
Lsm5	6	10.730	-1.100	-2.144	9.54E-05	7.53E-04		Ssc5d	7	0.541	1.315	2.488	5.61E-04	3.28E-03
Sptbn5	2	0.553	-1.016	-2.022	8.69E-05	6.99E-04		Cpa6	1	0.670	2.354	5.114	5.17E-04	3.07E-03
Gm13305	4	0.197	-3.527	-11.525	7.05E-05	5.88E-04		Sostdc1	12	1.391	1.703	3.256	5.17E-04	3.07E-03
Rskr	11	1.155	-1.547	-2.922	6.86E-05	5.74E-04		Prrg4	2	0.238	1.887	3.698	5.06E-04	3.01E-03

Tspan11	6	1.043	-1.110	-2.158	6.59E-05	5.54E-04		Igf1	10	0.840	1.012	2.016	5.00E-04	2.98E-03
Nup37	10	3.259	-1.048	-2.068	6.46E-05	5.46E-04		Clca3a1	3	0.405	2.144	4.421	4.97E-04	2.97E-03
Mis18a	16	4.969	-1.002	-2.003	6.21E-05	5.29E-04		Pkp2	16	2.943	1.050	2.071	4.83E-04	2.89E-03
Rdm1	11	6.523	-1.023	-2.032	6.17E-05	5.26E-04		Chrna2	14	0.373	1.816	3.522	4.65E-04	2.80E-03
Gm50253	17	0.692	-1.299	-2.460	5.15E-05	4.51E-04		Ppm1j	3	1.912	2.123	4.355	4.55E-04	2.75E-03
Ccdc84	9	1.602	-1.076	-2.108	5.02E-05	4.42E-04		Kcne2	16	0.776	3.433	10.803	4.40E-04	2.68E-03
Hba-a2	11	327.603	-1.155	-2.227	4.75E-05	4.20E-04		Slc18a2	19	0.242	2.092	4.262	4.07E-04	2.51E-03
Atoh7	10	0.949	-1.883	-3.687	4.25E-05	3.85E-04		Mecom	3	0.492	1.032	2.045	4.01E-04	2.49E-03
Mobp	9	149.552	-1.197	-2.293	4.18E-05	3.79E-04		Gpr101	X	2.267	1.238	2.358	3.87E-04	2.42E-03
Gm49333	16	0.173	-4.753	-26.955	3.97E-05	3.63E-04		Folr1	7	0.958	2.751	6.733	3.63E-04	2.29E-03
Sag	1	2.214	-1.175	-2.258	3.60E-05	3.34E-04		Gm9732	14	0.184	4.037	16.420	3.38E-04	2.17E-03
Gm14434	2	1.098	-1.913	-3.765	3.25E-05	3.07E-04		Six3	17	1.193	1.826	3.545	3.13E-04	2.03E-03
Proca1	11	2.839	-1.170	-2.251	3.13E-05	2.97E-04		Glra1	11	0.349	1.995	3.986	3.11E-04	2.02E-03
Coa4	7	3.778	-1.074	-2.105	2.80E-05	2.70E-04		P2ry1	3	0.729	1.236	2.356	3.03E-04	1.98E-03
Tmsb15b2	X	6.679	-1.255	-2.387	2.47E-05	2.41E-04		Gm49353	10	4.752	1.113	2.163	3.02E-04	1.98E-03
Ifit1bl1	19	2.309	-1.172	-2.254	2.32E-05	2.29E-04		Crhr2	6	0.621	1.482	2.793	3.00E-04	1.97E-03
Pdzph1	17	1.022	-1.192	-2.284	2.23E-05	2.22E-04		Shox2	3	0.151	4.922	30.316	2.86E-04	1.89E-03
Cyp2g1	7	0.524	-3.582	-11.972	2.00E-05	2.02E-04		Nppa	4	1.215	2.815	7.036	2.75E-04	1.83E-03
Hba-a1	11	178.809	-1.153	-2.223	1.92E-05	1.95E-04		Pcdhb6	18	1.024	1.068	2.096	2.71E-04	1.80E-03
Bcas1	2	78.413	-1.171	-2.251	1.60E-05	1.67E-04		Stac	9	1.083	1.276	2.421	2.68E-04	1.79E-03
Mbp	18	430.303	-1.091	-2.130	1.55E-05	1.63E-04		Nid2	14	1.065	1.030	2.042	2.54E-04	1.71E-03
Amn	12	4.397	-1.032	-2.045	1.55E-05	1.63E-04		Glra4	X	0.183	3.855	14.466	2.52E-04	1.70E-03
Tmsb15b1	X	6.223	-1.456	-2.743	1.54E-05	1.62E-04		Cdh3	8	0.218	2.379	5.201	2.52E-04	1.70E-03
Cenps	4	2.517	-1.228	-2.343	1.45E-05	1.54E-04		Insyn2a	7	2.619	1.035	2.049	2.44E-04	1.66E-03
Olfr78	7	0.111	-2.673	-6.378	1.34E-05	1.44E-04		Slc17a8	10	0.591	1.274	2.418	2.43E-04	1.65E-03
Gm10334	6	0.888	-2.599	-6.058	1.19E-05	1.30E-04		Rin3	12	0.771	1.039	2.054	2.30E-04	1.57E-03
Tefm	11	4.573	-1.065	-2.092	1.17E-05	1.28E-04		Epn3	11	0.525	1.916	3.773	2.14E-04	1.49E-03
Clec18a	8	1.026	-1.432	-2.699	1.14E-05	1.26E-04		Tubb6	18	1.831	1.661	3.162	2.09E-04	1.46E-03
G0s2	1	7.935	-1.055	-2.078	1.05E-05	1.17E-04		Cckar	5	0.091	4.360	20.538	2.08E-04	1.45E-03
Cd300c2	11	4.834	-1.222	-2.333	1.00E-05	1.13E-04		A2m	6	0.835	1.177	2.262	2.04E-04	1.43E-03
Pigbos1	9	13.772	-1.161	-2.237	8.31E-06	9.66E-05		Rbm20	19	1.051	1.044	2.062	2.02E-04	1.42E-03
Prcd	11	3.885	-1.293	-2.450	7.62E-06	8.94E-05		Mfrp	9	0.906	2.759	6.769	2.00E-04	1.41E-03
Gm6710	2	2.225	-1.253	-2.383	7.26E-06	8.60E-05		Crybg1	10	0.332	1.464	2.758	1.99E-04	1.40E-03
Ccl27b	4	2.163	-2.434	-5.405	5.45E-06	6.76E-05		Enpp3	10	0.147	2.078	4.223	1.90E-04	1.34E-03
Ccdc58	16	6.495	-1.085	-2.121	5.03E-06	6.35E-05		Epha2	4	0.735	1.270	2.412	1.86E-04	1.32E-03
Scx	15	2.199	-1.613	-3.058	3.60E-06	4.70E-05		Lama1	17	0.446	1.171	2.252	1.85E-04	1.31E-03
Gm5148	3	5.861	-1.011	-2.015	3.28E-06	4.36E-05		Tex15	8	0.210	1.636	3.109	1.82E-04	1.30E-03
Plb1	5	0.396	-1.753	-3.370	3.15E-06	4.22E-05		Ecel1	1	7.965	1.397	2.634	1.62E-04	1.18E-03
Kazald1	19	3.972	-1.335	-2.522	2.27E-06	3.18E-05		Frem2	3	0.361	1.144	2.210	1.62E-04	1.17E-03
Hilpda	6	6.604	-1.153	-2.223	2.21E-06	3.12E-05		Neb	2	0.132	1.449	2.729	1.51E-04	1.11E-03
Mdfic2	6	1.049	-2.025	-4.070	2.16E-06	3.07E-05		Gdnf	15	0.210	2.124	4.358	1.48E-04	1.09E-03
Cela1	15	2.345	-1.341	-2.533	1.61E-06	2.40E-05		Vdr	15	0.190	2.252	4.765	1.41E-04	1.04E-03
Evi2a	11	12.960	-1.040	-2.056	1.54E-06	2.31E-05		Tspan18	2	2.376	1.195	2.289	1.40E-04	1.04E-03
Efcab10	12	4.963	-1.564	-2.957	1.34E-06	2.06E-05		Filip1l	16	1.485	1.057	2.081	1.40E-04	1.04E-03
Dtl	1	1.322	-1.061	-2.086	1.29E-06	2.02E-05		Cyp26b1	6	3.077	1.030	2.042	1.23E-04	9.29E-04
Echdc2	4	5.909	-1.003	-2.004	1.05E-06	1.69E-05		Minar1	9	0.802	1.149	2.218	1.22E-04	9.28E-04
Ctla2a	13	6.182	-1.067	-2.094	7.90E-07	1.34E-05		Loxl1	9	1.440	1.105	2.151	1.20E-04	9.13E-04
Smim4	14	5.361	-1.196	-2.291	7.38E-07	1.28E-05		Pcdhb13	18	1.611	1.108	2.155	1.12E-04	8.62E-04
2310009B15Rik	1	10.617	-1.042	-2.059	7.15E-07	1.24E-05		Ntsr1	2	3.033	1.064	2.091	1.12E-04	8.58E-04
Hbb-bt	7	76.496	-1.614	-3.061	6.67E-07	1.17E-05		Dnah11	12	0.140	2.042	4.117	1.12E-04	8.57E-04
Mthfs	9	3.406	-1.466	-2.763	6.20E-07	1.10E-05		Drc7	8	1.009	1.908	3.754	1.07E-04	8.27E-04
Mthfsl	9	8.638	-1.093	-2.133	5.95E-07	1.07E-05		Lrrc55	2	5.433	1.112	2.162	1.05E-04	8.14E-04
Ormdl1	1	5.907	-1.081	-2.115	5.79E-07	1.04E-05		Npffr2	5	0.282	3.979	15.766	9.61E-05	7.57E-04
Plekhb1	7	128.788	-1.112	-2.162	5.58E-07	1.01E-05		Cspg4b	13	0.380	1.257	2.391	9.49E-05	7.51E-04
Psmg4	13	17.153	-1.091	-2.130	5.31E-07	9.68E-06		Pappa	4	0.108	2.309	4.954	9.43E-05	7.47E-04
Pcbd2	13	21.521	-1.039	-2.055	5.15E-07	9.46E-06		Mpz	1	0.219	4.216	18.585	9.19E-05	7.32E-04
Tyw5	1	5.236	-1.043	-2.061	4.27E-07	8.05E-06		Scube2	7	0.466	2.053	4.149	9.18E-05	7.31E-04
Gm14418	2	3.165	-1.320	-2.497	3.52E-07	6.89E-06		Fam184b	5	1.597	1.007	2.010	9.14E-05	7.28E-04
Sebox	11	2.533	-1.855	-3.616	3.33E-07	6.58E-06		Adamts9	6	0.694	1.190	2.282	8.39E-05	6.81E-04
Haus1	18	7.138	-1.096	-2.138	3.24E-07	6.42E-06		Sema5a	15	4.972	1.013	2.018	8.33E-05	6.77E-04
Agmat	4	1.568	-2.102	-4.292	2.89E-07	5.83E-06		Gdpd4	7	0.160	4.328	20.080	7.71E-05	6.35E-04
Rps20	4	57.768	-1.021	-2.030	2.24E-07	4.73E-06		Htr1d	4	0.901	1.560	2.949	7.05E-05	5.88E-04
Saysd1	14	5.773	-1.019	-2.027	2.13E-07	4.52E-06		Htr4	18	1.052	1.205	2.305	6.52E-05	5.50E-04
Shld1	2	6.828	-1.059	-2.083	1.77E-07	3.90E-06		Lgr6	1	1.955	2.060	4.169	6.34E-05	5.37E-04
Etnk2	1	3.174	-1.217	-2.324	1.74E-07	3.84E-06		Mab21l1	3	1.138	1.827	3.547	5.91E-05	5.08E-04
Oxld1	11	11.001	-1.201	-2.298	1.62E-07	3.61E-06		Emilin1	5	1.160	1.133	2.194	5.88E-05	5.06E-04
Gng11	6	15.850	-1.098	-2.140	1.10E-07	2.59E-06		Ccdc187	2	0.477	1.158	2.231	5.86E-05	5.05E-04
Thrsp	7	27.069	-1.144	-2.209	8.59E-08	2.09E-06		Il12rb2	6	0.150	4.186	18.205	5.52E-05	4.80E-04
Eef1b2	1	101.516	-1.050	-2.071	8.19E-08	2.01E-06		Svep1	4	0.195	1.702	3.254	5.35E-05	4.66E-04
Zfp931	2	5.877	-1.137	-2.199	7.50E-08	1.85E-06		Fbln2	6	1.527	1.049	2.069	5.03E-05	4.42E-04
S100a16	3	70.710	-1.025	-2.035	7.19E-08	1.79E-06		Slc6a3	13	0.439	2.823	7.075	4.77E-05	4.23E-04
Cox8a	19	952.702	-1.000	-2.001	7.02E-08	1.75E-06		Gng4	13	45.349	1.206	2.308	4.75E-05	4.20E-04
Cldn10	14	20.726	-1.016	-2.022	6.97E-08	1.74E-06		Lhx8	3	1.108	2.869	7.304	4.48E-05	4.01E-04
Ddit4l	3	8.382	-1.019	-2.026	5.97E-08	1.52E-06		Zfhx3	8	0.293	1.138	2.200	4.26E-05	3.85E-04

Mt3	8	426.586	-1.026	-2.037	5.02E-08	1.32E-06		Oprm1	10	0.276	1.526	2.880	4.19E-05	3.80E-04
Opalin	19	14.975	-1.064	-2.091	5.01E-08	1.32E-06		Lhx5	5	0.189	2.960	7.782	3.95E-05	3.62E-04
Fis1	5	179.651	-1.019	-2.027	4.95E-08	1.30E-06		Gcnt1	19	1.810	1.748	3.360	3.95E-05	3.62E-04
Tmed1	9	12.824	-1.013	-2.017	4.86E-08	1.28E-06		Crb1	1	0.429	1.355	2.558	3.91E-05	3.60E-04
Dpm3	3	19.997	-1.048	-2.068	4.80E-08	1.27E-06		Htr2c	X	12.536	1.051	2.072	3.85E-05	3.55E-04
Tyrobp	7	49.279	-1.101	-2.145	4.71E-08	1.25E-06		Rtl1	12	0.113	2.411	5.318	3.73E-05	3.45E-04
Lage3	X	8.604	-1.058	-2.082	4.55E-08	1.22E-06		Tafa3	3	0.220	3.446	10.896	3.71E-05	3.44E-04
Zswim7	11	13.747	-1.159	-2.233	4.16E-08	1.13E-06		Pcdha10	18	1.419	1.109	2.156	3.56E-05	3.32E-04
Nudcd2	11	10.596	-1.029	-2.041	3.99E-08	1.09E-06		Tpbgl	7	2.721	1.113	2.163	3.29E-05	3.10E-04
Hscb	5	9.706	-1.341	-2.534	3.81E-08	1.05E-06		Klhl1	14	0.693	1.364	2.574	3.27E-05	3.08E-04
Pdlim2	14	7.670	-1.129	-2.188	3.55E-08	9.99E-07		Grem1	2	0.812	1.679	3.202	2.69E-05	2.61E-04
Cox16	12	6.338	-1.011	-2.016	3.27E-08	9.31E-07		Adra2b	2	0.253	2.366	5.154	2.67E-05	2.58E-04
Vti1b	12	137.282	-1.062	-2.087	3.14E-08	8.98E-07		Shisa6	11	5.377	1.502	2.833	2.53E-05	2.46E-04
Nkain4	2	45.135	-1.001	-2.002	2.98E-08	8.62E-07		Slc35d3	10	2.602	1.809	3.505	2.48E-05	2.42E-04
Sec61b	4	37.398	-1.012	-2.017	2.73E-08	7.97E-07		Hspa1b	17	13.471	1.628	3.091	2.18E-05	2.17E-04
Selenow	7	1605.333	-1.053	-2.074	2.70E-08	7.93E-07		Igfbpl1	4	0.904	2.116	4.334	2.00E-05	2.02E-04
Ciao2a	9	25.636	-1.063	-2.089	2.64E-08	7.77E-07		Notch2	3	2.879	1.053	2.074	1.76E-05	1.81E-04
BC028528	3	6.361	-1.564	-2.956	2.61E-08	7.69E-07		Rassf4	6	1.813	1.171	2.251	1.63E-05	1.69E-04
Gm11808	4	868.926	-1.023	-2.033	2.48E-08	7.30E-07		Dsc3	18	0.583	1.455	2.742	1.49E-05	1.58E-04
Cox6b1	7	530.444	-1.009	-2.012	2.44E-08	7.23E-07		Nlrp10	7	0.190	3.398	10.543	1.43E-05	1.53E-04
Tnnc1	14	13.631	-1.578	-2.985	2.26E-08	6.77E-07		Pcdha6	18	1.454	1.089	2.127	1.43E-05	1.53E-04
Rpl27	11	201.738	-1.072	-2.103	2.23E-08	6.68E-07		Col5a2	1	0.720	1.220	2.329	1.42E-05	1.52E-04
Rpl7	1	319.809	-1.038	-2.054	2.17E-08	6.56E-07		Tenm2	11	8.758	1.125	2.181	1.34E-05	1.44E-04
Drap1	19	233.925	-1.017	-2.023	2.15E-08	6.52E-07		Chrm2	6	2.611	1.153	2.224	1.33E-05	1.43E-04
Mrpl54	10	58.985	-1.061	-2.087	1.93E-08	5.96E-07		Nkain3	4	0.969	1.332	2.517	1.30E-05	1.41E-04
Izumo4	10	13.011	-1.005	-2.006	1.77E-08	5.50E-07		Sh3rf2	18	1.059	1.704	3.257	1.27E-05	1.38E-04
Idnk	13	6.060	-1.021	-2.029	1.75E-08	5.44E-07		Myof	19	0.886	1.154	2.226	1.14E-05	1.26E-04
Rplp1	9	1415.181	-1.052	-2.073	1.58E-08	4.97E-07		Pcdha9	18	2.223	1.006	2.008	1.03E-05	1.16E-04
Uqcr11	10	456.561	-1.119	-2.173	1.51E-08	4.79E-07		Prok2	6	0.417	2.727	6.620	9.96E-06	1.13E-04
Hint1	11	275.140	-1.089	-2.128	1.43E-08	4.58E-07		Slit1	19	15.540	1.107	2.153	9.98E-06	1.13E-04
H2-T23	17	15.976	-1.069	-2.098	1.35E-08	4.42E-07		Cldn2	X	0.530	3.568	11.859	9.39E-06	1.07E-04
Rps28	17	249.919	-1.006	-2.008	1.33E-08	4.37E-07		Tchh	3	1.277	1.218	2.326	9.18E-06	1.05E-04
Bola3	6	13.695	-1.109	-2.156	1.33E-08	4.36E-07		Ttr	18	261.301	4.896	29.769	9.06E-06	1.04E-04
Zfp945	17	2.219	-1.100	-2.143	1.32E-08	4.36E-07		Gpr50	X	0.432	2.742	6.692	8.85E-06	1.02E-04
Ccdc107	4	39.563	-1.048	-2.068	1.20E-08	4.01E-07		Arhgap36	X	1.525	1.880	3.681	8.77E-06	1.01E-04
Rps17	7	510.139	-1.172	-2.253	1.19E-08	3.99E-07		Vangl1	3	0.688	1.178	2.262	8.75E-06	1.01E-04
Stac2	11	54.584	-1.089	-2.128	1.19E-08	3.99E-07		Itih2	2	2.214	1.190	2.281	7.62E-06	8.94E-05
Mrps16	14	12.617	-1.055	-2.078	9.54E-09	3.26E-07		Trp73	4	0.358	1.957	3.882	6.95E-06	8.27E-05
Nme3	17	35.438	-1.030	-2.042	8.60E-09	2.97E-07		Hspa1a	17	9.667	1.549	2.926	6.34E-06	7.69E-05
Rplp0	5	286.580	-1.068	-2.096	8.42E-09	2.92E-07		Abca4	3	0.524	1.494	2.816	6.21E-06	7.55E-05
Slirp	12	10.203	-1.053	-2.075	8.01E-09	2.79E-07		Dlx1	2	8.850	1.389	2.619	5.92E-06	7.25E-05
Hsd11b1	1	11.995	-1.496	-2.820	7.35E-09	2.59E-07		Scube3	17	0.505	1.639	3.115	5.59E-06	6.90E-05
Cstb	10	47.035	-1.018	-2.025	7.21E-09	2.55E-07		Rgs9	11	7.286	1.233	2.351	5.50E-06	6.81E-05
Mgst3	1	113.215	-1.001	-2.001	6.91E-09	2.47E-07		Oxtr	6	2.979	1.041	2.058	5.28E-06	6.59E-05
Rps6	4	559.255	-1.074	-2.106	6.71E-09	2.41E-07		Slc4a5	6	0.274	4.472	22.192	5.24E-06	6.55E-05
Med21	6	17.341	-1.076	-2.107	6.68E-09	2.40E-07		Zfhx4	3	0.700	1.043	2.060	4.74E-06	6.03E-05
Med31	11	11.717	-1.124	-2.179	6.22E-09	2.24E-07		Pde1c	6	0.784	1.011	2.016	4.53E-06	5.78E-05
Cox6a1	5	926.523	-1.086	-2.124	5.59E-09	2.04E-07		Acan	7	0.798	1.462	2.755	4.46E-06	5.70E-05
Cox7c	13	216.254	-1.165	-2.242	5.60E-09	2.04E-07		Meis1	11	1.040	1.699	3.247	4.27E-06	5.49E-05
Atp5o_2	16	281.631	-1.053	-2.074	5.30E-09	1.96E-07		Bend4	5	1.049	1.098	2.141	4.00E-06	5.18E-05
Mrps18c	5	29.719	-1.004	-2.005	5.29E-09	1.95E-07		Alx4	2	0.612	1.430	2.695	3.39E-06	4.48E-05
Rps9	7	228.943	-1.084	-2.120	5.26E-09	1.95E-07		Eln	5	2.472	1.145	2.212	3.03E-06	4.08E-05
Selenof	3	123.152	-1.138	-2.200	5.22E-09	1.93E-07		Thsd4	9	1.907	1.457	2.745	3.00E-06	4.05E-05
Nat8f1	6	12.161	-1.241	-2.363	4.96E-09	1.87E-07		Tnrc6b	15	4.076	1.017	2.024	2.63E-06	3.61E-05
Rpsa	9	327.177	-1.103	-2.148	4.89E-09	1.84E-07		Cntnap5c	17	1.534	1.337	2.526	2.62E-06	3.60E-05
Psmd10	X	17.127	-1.050	-2.071	4.48E-09	1.71E-07		Chrna3	9	0.268	2.978	7.877	2.25E-06	3.17E-05
Atp5h	11	426.107	-1.061	-2.087	4.43E-09	1.70E-07		Epha3	16	1.773	1.056	2.080	2.16E-06	3.07E-05
Dmac1	4	49.544	-1.002	-2.003	4.42E-09	1.69E-07		Sema3g	14	2.449	1.196	2.291	2.12E-06	3.02E-05
Tmem160	7	52.295	-1.023	-2.032	4.42E-09	1.69E-07		Fhdc1	3	0.928	1.305	2.470	1.99E-06	2.86E-05
Arl1	10	93.628	-1.097	-2.138	4.25E-09	1.65E-07		Kl	5	2.819	1.985	3.959	1.94E-06	2.80E-05
Hbb-bs	7	547.675	-1.903	-3.740	4.11E-09	1.60E-07		Tshz1	18	12.905	1.233	2.350	1.92E-06	2.77E-05
Tmem218	9	7.642	-1.062	-2.087	3.80E-09	1.49E-07		Sall3	18	2.012	1.397	2.633	1.79E-06	2.62E-05
Zfyve21	12	15.993	-1.109	-2.157	3.74E-09	1.48E-07		Hcn3	3	4.086	1.008	2.011	1.70E-06	2.51E-05
Rpl24	16	489.211	-1.088	-2.126	3.74E-09	1.47E-07		Tmem72	6	0.113	4.226	18.710	1.70E-06	2.51E-05
Nenf	1	113.198	-1.049	-2.069	3.54E-09	1.41E-07		Gabrq	X	0.626	2.025	4.071	1.69E-06	2.50E-05
Blvra	2	15.794	-1.022	-2.030	3.52E-09	1.41E-07		Prokr2	2	0.760	1.598	3.028	1.58E-06	2.37E-05
Romo1	2	75.128	-1.029	-2.041	3.33E-09	1.34E-07		Dll1	17	1.965	1.164	2.241	1.23E-06	1.93E-05
Oaz1	10	406.737	-1.054	-2.077	3.21E-09	1.31E-07		Igsf10	3	0.393	1.479	2.788	1.21E-06	1.91E-05
Rapgef4	2	252.576	-1.313	-2.484	3.08E-09	1.26E-07		Dcx	X	3.458	1.514	2.856	1.15E-06	1.82E-05
Atox1	11	90.711	-1.058	-2.081	3.05E-09	1.25E-07		Col3a1	1	1.318	1.415	2.667	1.05E-06	1.69E-05
Sertad1	7	14.140	-1.139	-2.203	3.02E-09	1.24E-07		Adamts19	18	0.221	4.327	20.068	1.03E-06	1.66E-05
Ndufs6	13	87.805	-1.073	-2.104	2.88E-09	1.19E-07		Thbs1	2	0.920	1.525	2.877	1.01E-06	1.63E-05
Rpl37	15	353.115	-1.167	-2.245	2.75E-09	1.14E-07		Thsd7b	1	1.374	1.098	2.141	1.00E-06	1.62E-05
Cd6	19	1.106	-2.057	-4.163	2.65E-09	1.11E-07		Cacna1e	1	14.741	1.072	2.102	9.77E-07	1.60E-05

Pts	9	29.160	-1.084	-2.120	2.46E-09	1.03E-07		Gbx1	5	0.547	2.637	6.221	9.60E-07	1.57E-05
1810009A15Rik	19	22.685	-1.057	-2.081	2.36E-09	9.99E-08		Tcf7l2	19	1.735	1.285	2.437	9.43E-07	1.56E-05
Rpl13	8	740.411	-1.118	-2.170	2.34E-09	9.92E-08		Il17rd	14	0.734	1.155	2.226	9.25E-07	1.53E-05
Cox14	15	36.994	-1.017	-2.024	2.19E-09	9.35E-08		Epb41l4a	18	1.498	1.411	2.659	9.25E-07	1.53E-05
Tmsb4x	X	703.242	-1.071	-2.100	2.17E-09	9.31E-08		Crispld2	8	1.531	1.264	2.402	9.18E-07	1.53E-05
Rpa3	6	12.687	-1.405	-2.647	1.99E-09	8.62E-08		Celsr2	3	21.444	1.097	2.139	9.15E-07	1.52E-05
Ramp3	11	11.320	-1.358	-2.564	1.99E-09	8.62E-08		Cep350	1	3.477	1.009	2.013	9.12E-07	1.52E-05
Eif3h	15	200.270	-1.073	-2.103	1.96E-09	8.53E-08		Baiap3	17	8.273	1.578	2.986	8.42E-07	1.42E-05
Rps3a1	3	582.464	-1.119	-2.173	1.91E-09	8.38E-08		Vipr2	12	1.853	2.813	7.028	8.28E-07	1.40E-05
Rpl14	9	333.108	-1.115	-2.165	1.89E-09	8.28E-08		Grin2b	6	13.969	1.327	2.509	8.08E-07	1.37E-05
Timm10	2	45.085	-1.038	-2.054	1.82E-09	8.01E-08		Prkca	11	25.259	1.009	2.012	7.88E-07	1.34E-05
Pigyl	9	43.882	-1.064	-2.090	1.82E-09	8.01E-08		Spata13	14	2.168	1.028	2.039	7.65E-07	1.31E-05
C1d	11	14.888	-1.003	-2.005	1.76E-09	7.82E-08		Isl1	13	0.785	2.524	5.751	6.22E-07	1.11E-05
Sar1b	11	58.194	-1.010	-2.014	1.76E-09	7.82E-08		Dock11	X	1.463	1.123	2.179	5.87E-07	1.05E-05
Ndufs5	4	183.378	-1.020	-2.028	1.75E-09	7.81E-08		Frem3	8	0.262	2.441	5.431	5.78E-07	1.04E-05
B9d1	11	20.153	-1.112	-2.161	1.69E-09	7.56E-08		Adra2a	19	6.285	1.035	2.049	5.01E-07	9.24E-06
Atp6v1g1	4	122.462	-1.001	-2.002	1.67E-09	7.52E-08		Ltbp2	12	0.232	3.372	10.354	4.91E-07	9.07E-06
Uqcrq	11	113.495	-1.180	-2.266	1.67E-09	7.52E-08		Gal	19	1.545	2.975	7.865	4.86E-07	9.00E-06
Ndufb7	8	198.793	-1.034	-2.047	1.56E-09	7.09E-08		Impg1	9	0.235	3.080	8.456	4.21E-07	7.97E-06
Tsen15	1	25.144	-1.032	-2.045	1.54E-09	7.02E-08		Col18a1	10	1.211	1.349	2.547	4.07E-07	7.75E-06
Ndufa2	18	180.058	-1.033	-2.046	1.55E-09	7.02E-08		Magel2	7	0.867	1.783	3.441	4.04E-07	7.70E-06
Nhp2	11	46.096	-1.025	-2.035	1.52E-09	6.98E-08		Slc9a4	1	1.264	1.726	3.309	3.96E-07	7.59E-06
Rps13	7	109.339	-1.173	-2.255	1.48E-09	6.80E-08		Peg3	7	27.883	1.056	2.079	3.80E-07	7.34E-06
Sec11c	18	92.855	-1.047	-2.067	1.43E-09	6.56E-08		Pcdhga4	18	6.345	1.024	2.033	3.71E-07	7.21E-06
Pard6a	8	25.608	-1.083	-2.118	1.28E-09	5.95E-08		Sdk1	5	0.876	1.052	2.074	3.54E-07	6.91E-06
Coa8	12	11.408	-1.028	-2.039	1.24E-09	5.84E-08		Hap1	11	65.482	1.095	2.135	3.52E-07	6.89E-06
Micos13	17	104.646	-1.088	-2.126	1.20E-09	5.71E-08		Gdpd5	7	6.761	1.118	2.170	3.33E-07	6.58E-06
Rbp4	19	14.722	-1.063	-2.089	1.14E-09	5.45E-08		Glp1r	17	0.405	2.197	4.585	3.13E-07	6.22E-06
Rbm7	9	15.390	-1.002	-2.003	1.13E-09	5.41E-08		Armcx4	X	7.815	1.039	2.055	3.09E-07	6.15E-06
Atp5j2	5	175.488	-1.173	-2.255	1.12E-09	5.39E-08		Kirrel	3	1.114	1.216	2.324	2.87E-07	5.79E-06
Atp6v0b	4	178.901	-1.053	-2.075	1.04E-09	5.03E-08		Alk	17	1.591	1.202	2.301	2.69E-07	5.50E-06
Avpi1	19	29.822	-1.142	-2.207	9.78E-10	4.74E-08		Syt10	15	7.904	1.325	2.505	2.36E-07	4.97E-06
Ssna1	2	44.439	-1.009	-2.013	9.67E-10	4.70E-08		Nhs	X	0.825	1.403	2.645	2.08E-07	4.42E-06
Gm14326	2	9.142	-1.038	-2.054	9.21E-10	4.50E-08		Tll1	8	0.711	1.806	3.498	2.06E-07	4.40E-06
Rbis	3	14.385	-1.094	-2.135	9.18E-10	4.49E-08		Ttc28	5	2.423	1.046	2.065	1.91E-07	4.14E-06
Alkbh7	17	25.442	-1.087	-2.124	9.13E-10	4.48E-08		Nav1	1	6.886	1.063	2.089	1.91E-07	4.14E-06
Mylk3	8	0.991	-1.678	-3.200	9.04E-10	4.45E-08		Arid5b	10	4.654	1.031	2.043	1.84E-07	4.03E-06
Coa6	8	26.874	-1.056	-2.079	9.04E-10	4.45E-08		Fstl5	3	7.281	1.391	2.623	1.78E-07	3.91E-06
Polr2i	7	26.405	-1.017	-2.024	8.84E-10	4.39E-08		Drd3	16	0.472	4.694	25.883	1.68E-07	3.74E-06
Zfand2b	1	16.906	-1.046	-2.064	8.28E-10	4.15E-08		Nrxn3	12	19.094	1.018	2.026	1.67E-07	3.73E-06
Hspe1	1	113.915	-1.024	-2.034	8.00E-10	4.06E-08		Tbx21	11	1.726	4.190	18.252	1.46E-07	3.31E-06
Kcnmb4	10	44.312	-1.001	-2.001	7.70E-10	3.95E-08		Creb3l2	6	2.248	1.012	2.017	1.31E-07	3.01E-06
Tmem126a	7	32.397	-1.064	-2.090	7.17E-10	3.72E-08		Stk32b	5	1.611	1.399	2.637	1.25E-07	2.88E-06
Gtf2a2	9	13.177	-1.124	-2.180	6.87E-10	3.60E-08		Lonrf2	1	29.059	1.001	2.001	1.18E-07	2.74E-06
Cox6c	15	193.352	-1.200	-2.297	6.85E-10	3.60E-08		Birc6	17	5.771	1.005	2.007	1.16E-07	2.71E-06
Ndufa11	17	107.437	-1.031	-2.044	6.90E-10	3.60E-08		Pcbp3	10	31.059	1.343	2.536	1.14E-07	2.65E-06
Rpl22l1	3	105.688	-1.150	-2.219	6.46E-10	3.41E-08		Tacr1	6	1.880	2.022	4.062	1.08E-07	2.54E-06
Glt8d2	10	7.325	-1.087	-2.124	6.43E-10	3.40E-08		Kcnk15	2	0.955	3.949	15.444	1.07E-07	2.54E-06
Ppih	4	9.200	-1.073	-2.103	5.59E-10	3.00E-08		Tnc	4	1.036	1.282	2.431	1.05E-07	2.48E-06
Cox17	16	17.833	-1.075	-2.106	5.60E-10	3.00E-08		Ntrk1	3	0.861	2.712	6.551	1.02E-07	2.43E-06
Rpl37a	1	371.977	-1.145	-2.211	5.51E-10	2.97E-08		Actn2	13	5.874	1.175	2.258	9.02E-08	2.19E-06
Prxl2b	4	123.097	-1.062	-2.088	5.45E-10	2.95E-08		Slc32a1	2	53.360	1.012	2.016	8.55E-08	2.09E-06
Bbip1	19	19.872	-1.027	-2.038	5.47E-10	2.95E-08		Lancl3	X	1.984	1.651	3.139	7.61E-08	1.87E-06
Mrpl46	7	35.530	-1.048	-2.068	5.35E-10	2.92E-08		Sp7	15	1.808	2.927	7.605	7.27E-08	1.80E-06
Trappc2l	8	32.411	-1.039	-2.054	5.03E-10	2.76E-08		Slc10a4	5	2.180	2.634	6.206	7.19E-08	1.79E-06
Atp6v1f	6	151.743	-1.008	-2.011	4.62E-10	2.55E-08		Trdn	10	0.231	3.301	9.855	6.63E-08	1.67E-06
Use1	8	69.645	-1.111	-2.160	4.59E-10	2.54E-08		Slc18a3	14	1.061	2.412	5.323	6.60E-08	1.66E-06
Sdhd	9	107.248	-1.031	-2.043	4.41E-10	2.45E-08		Sema3d	5	1.189	1.329	2.512	6.20E-08	1.57E-06
Mpc1	17	98.007	-1.036	-2.050	4.27E-10	2.39E-08		Scn2a	2	30.058	1.016	2.022	5.75E-08	1.47E-06
S100a1	3	146.851	-1.185	-2.273	4.17E-10	2.34E-08		Fry	5	12.431	1.131	2.190	5.72E-08	1.47E-06
Cryzl1	16	22.485	-1.026	-2.037	3.94E-10	2.23E-08		Hydin	8	0.167	1.885	3.693	5.47E-08	1.41E-06
Ss18l2	9	14.158	-1.137	-2.200	3.41E-10	1.96E-08		Inpp4b	8	1.345	1.148	2.216	5.27E-08	1.36E-06
Cox7a2	9	353.063	-1.214	-2.320	3.07E-10	1.78E-08		Pakap_3	4	5.907	1.036	2.050	5.12E-08	1.33E-06
Scg5	2	219.424	-1.090	-2.129	2.97E-10	1.73E-08		Tox3	8	5.340	1.086	2.124	5.02E-08	1.32E-06
Ciao2b	8	28.616	-1.106	-2.153	2.92E-10	1.71E-08		Dscaml1	9	11.138	1.066	2.093	4.68E-08	1.24E-06
Tomm7	5	74.592	-1.105	-2.151	2.86E-10	1.67E-08		Cntnap3	13	2.634	1.303	2.467	4.66E-08	1.24E-06
Rpl36al	12	68.249	-1.092	-2.131	2.82E-10	1.66E-08		Chrnb4	9	0.391	4.210	18.505	4.61E-08	1.23E-06
Mrps28	3	20.234	-1.150	-2.219	2.80E-10	1.65E-08		Nr2f2	7	7.107	1.149	2.218	4.58E-08	1.22E-06
Rgcc	14	33.555	-1.215	-2.321	2.79E-10	1.65E-08		Zfp366	13	1.440	1.277	2.424	4.45E-08	1.19E-06
Commd2	3	7.197	-1.114	-2.165	2.69E-10	1.60E-08		Foxo1	3	4.407	1.061	2.086	4.37E-08	1.18E-06
Dctn3	4	129.191	-1.040	-2.057	2.63E-10	1.58E-08		Gbx2	1	0.481	4.489	22.459	3.80E-08	1.05E-06
Rpl27a	7	359.921	-1.186	-2.276	2.47E-10	1.48E-08		Dlx5	6	7.388	1.589	3.009	3.77E-08	1.04E-06
Rps5	7	357.372	-1.202	-2.301	2.18E-10	1.32E-08		Igsf1	X	1.713	1.328	2.510	3.75E-08	1.04E-06
Robo3	9	1.638	-1.884	-3.690	2.12E-10	1.29E-08		Fndc1	17	0.949	1.589	3.008	3.56E-08	1.00E-06

Tbca	13	83.059	-1.057	-2.081	2.09E-10	1.28E-08		Ush1g	11	1.029	2.878	7.353	3.46E-08	9.75E-07
1110065P20Rik	4	40.675	-1.034	-2.048	2.05E-10	1.26E-08		Fam20c	5	17.038	1.018	2.026	3.41E-08	9.65E-07
Dgcr6	16	47.392	-1.106	-2.152	1.94E-10	1.20E-08		Sall1	8	7.930	1.188	2.279	3.36E-08	9.53E-07
Lamtor5	3	64.624	-1.049	-2.070	1.90E-10	1.17E-08		Pde3a	6	0.652	1.441	2.715	3.11E-08	8.93E-07
Mrpl20	4	56.533	-1.039	-2.055	1.78E-10	1.11E-08		Nxph4	10	5.932	1.669	3.179	3.08E-08	8.84E-07
Rps27rt	9	31.475	-1.860	-3.629	1.76E-10	1.10E-08		Krt9	11	4.107	1.151	2.220	3.06E-08	8.79E-07
Nop10	2	45.483	-1.042	-2.059	1.70E-10	1.07E-08		Dsg1c	18	0.252	3.704	13.028	2.89E-08	8.38E-07
Pdcd10	3	23.684	-1.087	-2.125	1.59E-10	1.01E-08		Zfp503	14	1.889	1.436	2.706	2.44E-08	7.23E-07
A430005L14Rik	4	25.514	-1.131	-2.191	1.45E-10	9.33E-09		AW551984	9	3.193	1.526	2.881	2.38E-08	7.07E-07
Supt4a	11	66.477	-1.081	-2.116	1.44E-10	9.33E-09		Npr1	3	2.291	2.130	4.378	2.35E-08	6.99E-07
Rpl10a	17	254.726	-1.187	-2.277	1.44E-10	9.33E-09		Gaa	11	95.077	1.051	2.072	2.27E-08	6.79E-07
Krt12	11	9.553	-1.345	-2.540	1.41E-10	9.17E-09		Gpr161	1	3.678	1.348	2.546	2.21E-08	6.66E-07
Mettl23	11	12.204	-1.126	-2.183	1.40E-10	9.14E-09		Atrx	X	8.169	1.075	2.107	2.20E-08	6.64E-07
Lin7b	7	78.436	-1.063	-2.089	1.26E-10	8.28E-09		Dlx2	2	5.256	1.704	3.259	2.13E-08	6.46E-07
Coa3	11	114.862	-1.007	-2.009	1.22E-10	8.11E-09		Ret	6	1.024	1.518	2.865	2.06E-08	6.31E-07
Ptpmt1	2	12.638	-1.107	-2.154	1.17E-10	7.86E-09		Cyp1b1	17	1.151	1.541	2.909	2.06E-08	6.31E-07
Rpl35	2	226.640	-1.303	-2.467	1.13E-10	7.62E-09		Aqp1	6	1.808	3.537	11.605	2.00E-08	6.16E-07
Rps23	13	428.004	-1.249	-2.376	1.07E-10	7.34E-09		Abca1	4	4.693	1.036	2.051	1.79E-08	5.53E-07
Serf1	13	11.032	-1.236	-2.356	1.06E-10	7.24E-09		Dgkg	16	22.447	1.085	2.121	1.66E-08	5.19E-07
Sft2d1	17	22.450	-1.149	-2.217	1.01E-10	6.99E-09		Pag1	3	3.001	1.040	2.056	1.64E-08	5.15E-07
Atpif1	4	449.563	-1.152	-2.222	9.61E-11	6.64E-09		Sema6a	18	4.452	1.018	2.025	1.64E-08	5.13E-07
Rpl31	1	292.055	-1.259	-2.393	9.47E-11	6.60E-09		Sv2a	3	113.223	1.030	2.042	1.52E-08	4.80E-07
Pop5	5	34.957	-1.152	-2.222	8.78E-11	6.18E-09		Wnt5a	14	1.849	1.241	2.363	1.50E-08	4.79E-07
Rpl36a	X	299.385	-1.152	-2.222	8.77E-11	6.18E-09		St3gal1	15	7.302	1.003	2.004	1.44E-08	4.61E-07
Rpl11	4	410.997	-1.244	-2.368	8.65E-11	6.14E-09		Grik3	4	10.474	1.016	2.022	1.42E-08	4.58E-07
Vcpkmt	12	16.446	-1.167	-2.245	8.40E-11	5.98E-09		Zfp608	18	4.838	1.186	2.276	1.42E-08	4.58E-07
Gng10	4	53.701	-1.138	-2.201	6.98E-11	4.99E-09		Runx1t1	4	2.654	1.048	2.068	1.41E-08	4.57E-07
Rpl36	17	538.114	-1.326	-2.506	6.97E-11	4.99E-09		Cemip2	19	2.214	1.079	2.113	1.41E-08	4.57E-07
Gls2	10	8.821	-1.052	-2.074	6.77E-11	4.88E-09		Sp9	2	3.199	1.863	3.637	1.35E-08	4.42E-07
Tma7	9	110.473	-1.052	-2.073	6.37E-11	4.61E-09		Ylpm1	12	14.427	1.151	2.220	1.35E-08	4.42E-07
Psmb10	8	36.444	-1.087	-2.124	6.21E-11	4.51E-09		Kcnd2	6	17.589	1.100	2.144	1.32E-08	4.35E-07
Rack1	11	248.145	-1.215	-2.321	5.75E-11	4.19E-09		Foxp2	6	1.287	1.260	2.395	1.27E-08	4.20E-07
Rpl38	11	493.499	-1.251	-2.380	5.72E-11	4.19E-09		Pde11a	2	1.299	1.479	2.787	1.26E-08	4.17E-07
Polr2e	10	81.028	-1.008	-2.011	5.53E-11	4.08E-09		Slc8a1	17	10.306	1.215	2.321	1.21E-08	4.01E-07
Arhgdig	17	92.215	-1.030	-2.043	5.54E-11	4.08E-09		Prkg1	19	1.700	1.228	2.343	1.16E-08	3.90E-07
Rnf7	9	85.667	-1.062	-2.088	5.39E-11	4.02E-09		Abl2	1	8.261	1.045	2.064	1.06E-08	3.57E-07
Acyp2	11	29.454	-1.167	-2.245	5.01E-11	3.81E-09		Abi3bp	16	1.797	1.789	3.457	1.04E-08	3.51E-07
Mrps33	6	63.453	-1.031	-2.044	4.10E-11	3.14E-09		Pcdhgb6	18	8.261	1.037	2.052	9.96E-09	3.39E-07
Mtln	2	41.715	-1.162	-2.238	3.78E-11	2.93E-09		Nexmif	X	3.948	1.041	2.058	9.18E-09	3.15E-07
Rps8	4	622.285	-1.227	-2.341	3.76E-11	2.93E-09		Ace	11	5.447	1.209	2.312	9.09E-09	3.13E-07
Pstk	7	13.656	-1.196	-2.291	3.76E-11	2.93E-09		Pcdhga1	18	2.741	1.067	2.096	8.10E-09	2.82E-07
Rps27a	11	434.982	-1.313	-2.484	3.79E-11	2.93E-09		Pcdhga10	18	5.126	1.066	2.093	7.97E-09	2.79E-07
Dpm1	2	6.420	-1.169	-2.249	3.68E-11	2.90E-09		Atp8a2	14	4.454	1.065	2.092	7.41E-09	2.61E-07
Lsm1	8	10.382	-1.104	-2.150	3.55E-11	2.82E-09		Itga4	2	2.056	1.104	2.150	7.21E-09	2.55E-07
Naa38	11	75.298	-1.122	-2.176	3.39E-11	2.70E-09		Gad1	2	102.059	1.189	2.280	5.94E-09	2.15E-07
Nme2	11	59.952	-1.226	-2.339	3.19E-11	2.56E-09		Kcnb2	1	1.928	1.276	2.422	5.73E-09	2.08E-07
Arpp19	9	81.280	-1.143	-2.209	3.02E-11	2.46E-09		Brd4	17	12.329	1.028	2.039	5.46E-09	2.00E-07
Mpc2	1	107.807	-1.081	-2.116	2.35E-11	1.92E-09		Cracd	5	6.737	1.066	2.094	5.22E-09	1.93E-07
Ndufb6	4	126.344	-1.118	-2.171	2.32E-11	1.92E-09		Cntnap5a	1	4.802	1.047	2.066	5.19E-09	1.93E-07
Bok	1	41.610	-1.117	-2.169	2.30E-11	1.90E-09		Atp2b4	1	34.293	1.131	2.190	5.14E-09	1.92E-07
Mrpl12	11	87.780	-1.117	-2.170	2.29E-11	1.90E-09		Tmem255a	X	10.525	1.176	2.260	4.75E-09	1.79E-07
Ndufb2	6	23.583	-1.099	-2.142	2.27E-11	1.90E-09		Tmem131	1	10.427	1.050	2.071	4.68E-09	1.78E-07
S100a6	3	17.280	-1.342	-2.536	2.24E-11	1.89E-09		Ankfn1	11	1.145	1.627	3.089	4.62E-09	1.76E-07
Mrpl41	2	74.708	-1.105	-2.151	2.23E-11	1.88E-09		F5	1	0.554	3.393	10.505	4.35E-09	1.68E-07
Smim26	2	39.871	-1.199	-2.296	2.17E-11	1.84E-09		Slco5a1	1	0.449	2.318	4.987	4.24E-09	1.64E-07
Bri3	5	50.339	-1.127	-2.184	2.00E-11	1.71E-09		Slc6a9	4	12.981	1.066	2.093	3.90E-09	1.53E-07
Bnip3	7	87.361	-1.084	-2.120	1.93E-11	1.67E-09		Camk2d	3	14.373	1.127	2.184	3.63E-09	1.44E-07
Serp2	14	132.735	-1.122	-2.177	1.92E-11	1.67E-09		Glg1	8	36.124	1.161	2.236	3.64E-09	1.44E-07
Rps27l	9	28.486	-1.268	-2.408	1.70E-11	1.53E-09		Slc8a3	12	6.143	1.195	2.290	3.58E-09	1.42E-07
Rps16	7	297.442	-1.282	-2.432	1.63E-11	1.47E-09		Car12	9	6.261	1.124	2.180	3.40E-09	1.36E-07
Coq2	5	46.236	-1.110	-2.158	1.57E-11	1.43E-09		Trim62	4	6.919	1.099	2.142	3.38E-09	1.36E-07
Chchd6	6	91.373	-1.069	-2.099	1.43E-11	1.33E-09		Diaph2	X	2.783	1.048	2.068	3.34E-09	1.35E-07
Ndufaf5	2	45.218	-1.140	-2.204	1.41E-11	1.32E-09		Epha5	5	10.794	1.025	2.035	3.31E-09	1.34E-07
Rps10	17	203.467	-1.302	-2.466	1.39E-11	1.31E-09		Gprin3	6	1.574	1.206	2.307	3.26E-09	1.32E-07
Ramp1	1	45.692	-1.194	-2.288	1.27E-11	1.21E-09		Cep170	1	7.698	1.072	2.103	3.11E-09	1.27E-07
Fau	19	456.742	-1.273	-2.417	1.20E-11	1.15E-09		Patj	4	1.161	1.541	2.911	3.08E-09	1.26E-07
Ssr4	X	67.560	-1.129	-2.188	1.17E-11	1.13E-09		Bmp6	13	5.423	1.029	2.041	2.90E-09	1.20E-07
Polr2k	15	34.655	-1.264	-2.402	1.13E-11	1.10E-09		Pbx3	2	9.370	1.951	3.865	2.54E-09	1.06E-07
Rpl39	X	221.883	-1.249	-2.376	1.02E-11	9.95E-10		Pcdh8	14	15.444	1.130	2.188	2.50E-09	1.05E-07
Gm13304	4	27.472	-1.284	-2.436	9.95E-12	9.74E-10		Slc5a3	16	2.627	1.378	2.599	2.49E-09	1.05E-07
Tmem42	9	15.537	-1.188	-2.278	9.87E-12	9.72E-10		Tacr3	3	1.164	1.712	3.276	2.44E-09	1.03E-07
Gpx1	9	135.875	-1.226	-2.339	8.59E-12	8.51E-10		Csmd3	15	1.777	1.188	2.278	2.20E-09	9.41E-08
Pradc1	6	14.909	-1.234	-2.353	8.15E-12	8.11E-10		Smoc1	12	9.338	1.119	2.172	2.11E-09	9.09E-08
Rpl19	11	944.560	-1.300	-2.463	8.09E-12	8.10E-10		Zfp462	4	4.285	1.147	2.214	2.02E-09	8.74E-08

Disease Models & Mechanisms • Supplementary information

Rpl26	11	681.432	-1.341	-2.533	7.60E-12	7.69E-10		Atg2b	12	6.801	1.053	2.074	2.00E-09	8.64E-08
Bud31	5	65.330	-1.125	-2.181	7.44E-12	7.61E-10		Inpp5j	11	15.085	1.316	2.489	1.95E-09	8.52E-08
Tmem208	8	20.091	-1.234	-2.352	7.37E-12	7.61E-10		Dgkk	X	0.486	2.037	4.103	1.77E-09	7.82E-08
Smim36	11	10.048	-1.164	-2.241	7.43E-12	7.61E-10		Klhl30	1	0.859	4.112	17.288	1.69E-09	7.56E-08
Ndufb8	19	126.274	-1.286	-2.439	6.73E-12	7.05E-10		Hspg2	4	0.971	1.431	2.696	1.69E-09	7.56E-08
Vps29	5	51.228	-1.113	-2.163	6.56E-12	6.90E-10		Chd6	2	7.056	1.097	2.139	1.42E-09	6.53E-08
Rps7	12	430.269	-1.310	-2.480	5.76E-12	6.14E-10		Slc9a1	4	12.799	1.031	2.043	1.31E-09	6.07E-08
Bola2	7	28.810	-1.214	-2.319	5.68E-12	6.08E-10		Myh9	15	12.502	1.078	2.111	1.29E-09	5.99E-08
Atp5l	9	489.142	-1.213	-2.318	5.58E-12	6.04E-10		Amigo2	15	3.777	1.251	2.380	1.26E-09	5.89E-08
Sirt3	7	51.832	-1.144	-2.210	5.46E-12	5.95E-10		Shisa3	5	2.134	4.181	18.143	1.25E-09	5.86E-08
Rps11	7	537.293	-1.310	-2.479	5.28E-12	5.80E-10		Zfp618	4	0.995	1.331	2.516	1.24E-09	5.84E-08
Cox4i1	8	1125.198	-1.321	-2.499	5.14E-12	5.68E-10		Pdgfra	5	8.418	1.023	2.032	1.24E-09	5.84E-08
Ndufa4	6	589.078	-1.254	-2.384	5.08E-12	5.63E-10		Parm1	5	16.911	1.124	2.179	1.13E-09	5.39E-08
Mrpl13	15	34.701	-1.188	-2.278	4.82E-12	5.39E-10		Myh10	11	32.158	1.118	2.171	9.30E-10	4.53E-08
Sytl2	7	7.067	-1.149	-2.218	4.54E-12	5.13E-10		Cachd1	4	3.762	1.115	2.166	8.65E-10	4.31E-08
Ndufs4	13	41.817	-1.127	-2.183	4.28E-12	4.87E-10		Strn	17	8.427	1.020	2.028	8.67E-10	4.31E-08
Rps15	10	1118.562	-1.361	-2.569	4.11E-12	4.71E-10		Chat	14	1.677	2.596	6.047	8.30E-10	4.15E-08
Nt5c	11	46.877	-1.126	-2.182	4.11E-12	4.71E-10		Kirrel3	9	13.597	1.029	2.041	8.19E-10	4.14E-08
Bloc1s2	19	51.503	-1.151	-2.220	4.07E-12	4.71E-10		Pde10a	17	15.985	1.190	2.281	8.01E-10	4.06E-08
Rps12	10	409.667	-1.446	-2.725	4.01E-12	4.70E-10		Cntnap4	8	6.841	1.157	2.230	7.89E-10	4.03E-08
Fmc1	6	71.237	-1.352	-2.553	3.77E-12	4.48E-10		Astn1	1	40.418	1.104	2.149	7.77E-10	3.97E-08
Mrpl28	17	76.515	-1.226	-2.340	3.75E-12	4.48E-10		Prlr	15	0.353	2.565	5.917	7.42E-10	3.82E-08
Rps19	7	163.352	-1.345	-2.540	3.56E-12	4.31E-10		Shisa8	15	5.827	2.872	7.322	7.25E-10	3.75E-08
Dkkl1	7	22.477	-1.228	-2.342	3.18E-12	3.93E-10		Adamtsl1	4	1.104	1.325	2.505	7.07E-10	3.68E-08
Gm21586	4	14.680	-1.977	-3.937	3.06E-12	3.82E-10		Disp2	2	40.987	1.172	2.253	6.88E-10	3.60E-08
Snrpg	6	36.757	-1.275	-2.420	3.03E-12	3.82E-10		Zswim5	4	3.940	1.121	2.175	6.35E-10	3.37E-08
Atp5k	5	409.610	-1.260	-2.395	2.71E-12	3.45E-10		Dchs2	3	0.396	2.191	4.565	6.04E-10	3.22E-08
Acot13	13	140.840	-1.227	-2.341	2.70E-12	3.45E-10		Heatr5b	17	8.180	1.090	2.129	5.77E-10	3.08E-08
Atp5md	19	83.141	-1.373	-2.590	2.57E-12	3.31E-10		Map3k1	13	2.409	1.741	3.342	5.31E-10	2.91E-08
Psma7	2	129.006	-1.150	-2.219	2.30E-12	3.03E-10		Calb2	8	52.968	2.099	4.283	4.41E-10	2.45E-08
Rpain	11	10.116	-1.399	-2.638	2.28E-12	3.03E-10		Fmod	1	12.626	1.148	2.217	4.03E-10	2.27E-08
Snrnp25	11	32.574	-1.251	-2.380	2.18E-12	2.91E-10		Sp8	12	2.339	3.498	11.298	4.01E-10	2.27E-08
Cebpzos	17	24.619	-1.222	-2.333	2.08E-12	2.82E-10		Dnm3	1	17.933	1.090	2.128	3.92E-10	2.23E-08
Ovol2	2	4.309	-1.921	-3.787	2.05E-12	2.81E-10		Numa1	7	12.523	1.102	2.146	3.80E-10	2.17E-08
Gm14295	2	8.166	-1.213	-2.318	2.04E-12	2.81E-10		Kit	5	12.124	1.124	2.179	3.67E-10	2.10E-08
Ndufb1	12	82.045	-1.254	-2.385	2.03E-12	2.81E-10		Tpr	1	13.717	1.032	2.044	3.53E-10	2.03E-08
Rpl5	5	280.651	-1.388	-2.616	1.72E-12	2.47E-10		Notch3	17	2.380	1.070	2.100	3.39E-10	1.96E-08
Tmem141	2	25.858	-1.221	-2.332	1.53E-12	2.23E-10		Cacng5	11	11.277	2.262	4.796	2.79E-10	1.65E-08
Rpl9-ps6	19	17.573	-1.960	-3.889	1.26E-12	1.88E-10		Zc3h13	14	9.941	1.219	2.328	2.69E-10	1.60E-08
1810037I17Rik	3	69.280	-1.270	-2.411	1.17E-12	1.77E-10		Kctd12	14	19.037	1.068	2.097	2.32E-10	1.40E-08
Gpx4	10	490.006	-1.344	-2.538	1.15E-12	1.76E-10		Prrc2c	1	13.338	1.276	2.421	2.23E-10	1.35E-08
Medag	5	8.542	-1.413	-2.663	1.13E-12	1.74E-10		Rrbp1	2	9.715	1.036	2.050	2.07E-10	1.27E-08
Rps24	14	339.149	-1.443	-2.720	1.00E-12	1.56E-10		Sorcs2	5	10.146	1.061	2.086	1.87E-10	1.16E-08
Immp1l	2	26.872	-1.241	-2.363	9.91E-13	1.55E-10		Zfp516	18	2.957	1.182	2.268	1.75E-10	1.10E-08
Gm2000	1	272.523	-1.309	-2.477	8.82E-13	1.41E-10		L1cam	X	31.708	1.119	2.172	1.67E-10	1.06E-08
Rps3	7	333.208	-1.408	-2.654	7.84E-13	1.26E-10		Gprasp1	X	83.709	1.195	2.290	1.66E-10	1.05E-08
Chchd1	14	59.108	-1.211	-2.314	7.64E-13	1.24E-10		Grm4	17	7.086	1.574	2.978	1.53E-10	9.74E-09
Mrpl14	17	41.175	-1.216	-2.324	7.05E-13	1.16E-10		Scgn	13	2.777	4.115	17.323	1.50E-10	9.63E-09
Tmem242	17	53.182	-1.225	-2.337	6.77E-13	1.13E-10		Tnr	1	15.720	1.241	2.364	1.49E-10	9.56E-09
Ndufb9	15	531.951	-1.379	-2.600	6.62E-13	1.11E-10		Fras1	5	1.305	1.211	2.316	1.39E-10	9.10E-09
Arhgap15	2	3.968	-1.358	-2.563	6.32E-13	1.07E-10		Lamb1	12	3.703	1.290	2.446	1.24E-10	8.19E-09
1110032F04Rik	3	7.229	-1.261	-2.397	6.03E-13	1.03E-10		Doc2g	19	32.343	4.075	16.856	1.20E-10	8.01E-09
Churc1	12	63.499	-1.206	-2.308	5.74E-13	9.93E-11		Pnma8b	7	125.206	1.144	2.210	1.19E-10	7.96E-09
Pet100	8	21.997	-1.334	-2.522	5.68E-13	9.91E-11		Igf2r	17	4.580	1.146	2.213	1.15E-10	7.72E-09
Timm10b	7	11.252	-1.171	-2.252	5.51E-13	9.70E-11		Slc7a14	3	15.630	1.245	2.371	1.11E-10	7.52E-09
Cd302	2	18.749	-1.320	-2.497	5.09E-13	9.13E-11		Pcdhga2	18	4.896	1.187	2.277	1.10E-10	7.51E-09
Atp5mpl	12	162.351	-1.242	-2.365	4.88E-13	8.85E-11		Hectd1	12	14.164	1.183	2.271	9.57E-11	6.64E-09
Ndufaf2	13	8.459	-1.301	-2.465	4.59E-13	8.59E-11		Ackr1	1	19.072	1.066	2.093	9.46E-11	6.60E-09
Rpl32	6	331.435	-1.415	-2.667	3.20E-13	6.10E-11		Sox1	8	5.342	1.141	2.205	9.10E-11	6.38E-09
Camk2n1	4	865.622	-1.389	-2.619	3.15E-13	6.06E-11		Col25a1	3	6.523	1.328	2.510	5.51E-11	4.08E-09
gene:ENSMUSG00000115423	2	27.714	-1.248	-2.375	2.52E-13	4.97E-11		Plce1	19	1.959	1.117	2.168	5.38E-11	4.02E-09
Guk1	11	154.448	-1.277	-2.424	2.45E-13	4.88E-11		Dpysl3	18	10.024	1.088	2.126	5.26E-11	3.95E-09
Car8	4	5.822	-1.493	-2.815	2.11E-13	4.25E-11		Slc5a7	17	1.223	2.377	5.194	5.08E-11	3.83E-09
Rpl13a	7	798.360	-1.481	-2.792	2.10E-13	4.25E-11		Unc5c	3	3.596	1.209	2.311	5.05E-11	3.82E-09
Dctpp1	7	24.670	-1.354	-2.557	1.92E-13	3.94E-11		Nynrin	14	2.230	1.202	2.300	4.15E-11	3.17E-09
Rpl41	10	1279.109	-1.450	-2.732	1.85E-13	3.85E-11		Cpd	11	13.941	1.084	2.120	4.07E-11	3.13E-09
Cd34	1	34.742	-1.162	-2.238	1.71E-13	3.59E-11		Srgap1	10	2.737	1.207	2.309	3.78E-11	2.93E-09
Rpl34	3	121.667	-1.544	-2.916	1.57E-13	3.34E-11		Thsd7a	6	4.024	1.384	2.610	3.27E-11	2.62E-09
Rpl35a	16	399.006	-1.491	-2.810	1.43E-13	3.11E-11		Pcdhgb5	18	2.857	1.281	2.429	3.13E-11	2.53E-09
Bbln	2	70.642	-1.338	-2.529	1.20E-13	2.65E-11		Scn3a	2	4.356	1.228	2.343	3.06E-11	2.48E-09
Rps18	17	488.375	-1.436	-2.705	1.19E-13	2.65E-11		Aebp1	11	9.651	1.268	2.409	2.34E-11	1.92E-09
Uqcrh	4	646.578	-1.430	-2.695	1.08E-13	2.45E-11		Ankrd63	2	11.012	1.294	2.452	2.06E-11	1.76E-09
Elof1	9	43.907	-1.235	-2.354	9.17E-14	2.14E-11		Nectin1	9	12.922	1.189	2.280	1.96E-11	1.69E-09
Cep20	16	46.584	-1.279	-2.427	4.74E-14	1.13E-11		Raver1	9	7.821	1.349	2.548	1.89E-11	1.65E-09

Mien1	11	139.140	-1.195	-2.289	4.53E-14	1.11E-11		Lamc1	1	5.222	1.209	2.312	1.81E-11	1.59E-09
Mrpl33	5	15.113	-1.428	-2.691	4.18E-14	1.04E-11		Cdh4	2	7.614	1.255	2.387	1.79E-11	1.57E-09
Cox7b	X	298.286	-1.385	-2.612	3.50E-14	8.90E-12		Th	7	8.572	3.884	14.764	1.75E-11	1.54E-09
Rps21	2	901.784	-1.534	-2.895	2.90E-14	7.92E-12		Map1b	13	70.502	1.411	2.660	1.73E-11	1.54E-09
Rps25	9	288.305	-1.451	-2.734	2.34E-14	6.59E-12		Syt6	3	6.522	2.039	4.109	1.71E-11	1.53E-09
Uqcr10	11	399.904	-1.335	-2.523	2.32E-14	6.59E-12		Wfs1	5	54.162	1.268	2.407	1.63E-11	1.47E-09
Rps27	3	554.984	-1.515	-2.857	1.54E-14	4.77E-12		Cacna1c	6	5.791	1.320	2.496	1.56E-11	1.43E-09
Vip	10	37.569	-1.281	-2.429	1.56E-14	4.77E-12		Slc29a4	5	8.308	1.176	2.259	1.46E-11	1.35E-09
Pfdn5	15	228.147	-1.487	-2.803	1.29E-14	4.21E-12		Clic6	16	2.452	2.505	5.677	1.45E-11	1.34E-09
Rpl9	5	304.025	-1.553	-2.935	9.22E-15	3.25E-12		Pcdhga5	18	5.840	1.150	2.219	1.42E-11	1.33E-09
Ndufa7	17	294.099	-1.359	-2.565	6.87E-15	2.52E-12		Zswim6	13	5.284	1.208	2.311	1.31E-11	1.24E-09
Ift20	11	57.388	-1.334	-2.520	6.56E-15	2.45E-12		Fat1	8	4.625	1.386	2.614	8.07E-12	8.10E-10
Rpl12	2	382.798	-1.534	-2.895	5.33E-15	2.07E-12		Frmd7	X	3.510	3.994	15.938	7.52E-12	7.65E-10
Gm20878	4	9.870	-1.981	-3.949	4.79E-15	2.04E-12		Cacna2d2	9	8.884	1.139	2.202	7.00E-12	7.29E-10
Cst6	19	2.805	-1.570	-2.970	4.43E-15	1.93E-12		Ntn1	11	2.991	1.308	2.476	5.98E-12	6.33E-10
Lsm7	10	37.524	-1.291	-2.447	4.19E-15	1.87E-12		Lamc3	2	1.754	1.403	2.645	5.61E-12	6.05E-10
Ccl21d	4	7.138	-3.266	-9.618	3.29E-15	1.51E-12		Slit3	11	9.393	1.399	2.637	4.77E-12	5.36E-10
Rpl17	18	549.320	-1.584	-2.997	2.77E-15	1.30E-12		Otof	5	4.388	1.430	2.694	4.10E-12	4.71E-10
AI413582	17	56.494	-1.400	-2.639	1.49E-15	7.17E-13		Col1a2	6	5.163	1.177	2.262	3.87E-12	4.58E-10
Rps14	18	414.518	-1.605	-3.043	7.52E-16	3.94E-13		Sez6	11	64.844	1.267	2.407	3.60E-12	4.34E-10
Cops9	1	106.297	-1.396	-2.631	7.20E-16	3.92E-13		Shisa9	16	16.460	1.201	2.299	3.47E-12	4.23E-10
Sec61g	11	33.549	-1.415	-2.667	7.28E-16	3.92E-13		Gad2	2	42.753	1.415	2.667	3.42E-12	4.21E-10
Atp5e	2	453.391	-1.485	-2.800	6.28E-16	3.60E-13		Insyn2b	11	1.165	2.632	6.198	3.06E-12	3.82E-10
Cpne9	6	14.920	-1.451	-2.735	1.88E-16	1.23E-13		Dchs1	7	2.185	1.274	2.419	2.54E-12	3.30E-10
Ndufb4	16	180.611	-1.433	-2.700	1.71E-16	1.16E-13		Strip2	6	6.451	1.268	2.408	2.43E-12	3.18E-10
Uqcc2	17	267.408	-1.501	-2.830	8.67E-17	6.62E-14		Pgap1	1	3.317	1.388	2.618	2.12E-12	2.86E-10
Gng13	17	89.320	-1.521	-2.870	8.14E-17	6.48E-14		Kcna5	6	3.759	1.802	3.488	2.00E-12	2.79E-10
Mrpl53	6	62.839	-1.383	-2.608	3.87E-17	3.38E-14		Ndnf	6	3.652	1.670	3.181	1.89E-12	2.67E-10
Tmem256	11	124.314	-1.525	-2.879	3.59E-17	3.38E-14		Erich3	3	2.812	1.300	2.463	1.76E-12	2.50E-10
Timm8b	9	262.456	-1.442	-2.716	2.20E-17	2.28E-14		Dhx9	1	22.554	1.174	2.256	1.61E-12	2.32E-10
Igfbp6	15	53.281	-1.411	-2.660	2.24E-17	2.28E-14		C4b	17	7.788	1.307	2.475	1.41E-12	2.06E-10
Acyp1	12	19.279	-1.507	-2.842	1.05E-17	1.20E-14		Bgn	X	11.680	1.265	2.404	1.29E-12	1.90E-10
Tomm5	4	71.549	-1.515	-2.858	7.02E-18	8.57E-15		Zic1	9	4.162	1.595	3.021	1.25E-12	1.88E-10
Znhit3	11	40.763	-1.554	-2.937	2.27E-18	3.19E-15		Tenm4	7	8.792	1.291	2.446	9.17E-13	1.45E-10
Rspo1	4	6.578	-2.173	-4.509	1.78E-18	2.72E-15		Grin3a	4	9.822	1.299	2.460	7.53E-13	1.23E-10
Gm13306	4	12.675	-2.616	-6.129	6.50E-22	1.70E-18		Cdhr1	14	20.545	3.488	11.219	5.26E-13	9.36E-11
Pvalb	15	74.155	-1.995	-3.987	9.23E-25	5.52E-21		Soga1	2	4.763	1.427	2.689	4.85E-13	8.85E-11
Myl4	11	16.799	-1.928	-3.807	2.23E-28	2.04E-24		Dpysl5	5	7.088	1.239	2.360	4.79E-13	8.85E-11
Ccl27a	4	70.635	-1.850	-3.604	5.75E-32	1.05E-27		Epha6	16	8.139	1.452	2.736	3.72E-13	7.03E-11
								Crybg3	16	1.179	1.499	2.827	2.84E-13	5.53E-11
								Nid1	13	2.403	1.519	2.866	1.49E-13	3.21E-11
								Zfp804a	2	5.302	1.294	2.453	9.41E-14	2.16E-11
								Pcdhga9	18	5.344	1.422	2.679	9.22E-14	2.14E-11
								Gpr149	3	1.051	2.466	5.524	4.65E-14	1.12E-11
								Myo16	8	10.397	1.933	3.818	3.80E-14	9.54E-12
								Kcnd3	3	12.461	1.416	2.669	3.48E-14	8.90E-12
								Pappa2	1	0.885	2.219	4.656	3.32E-14	8.80E-12
								Ms4a15	19	6.186	7.645	200.113	3.36E-14	8.80E-12
								Ntng1	3	7.106	1.371	2.587	3.00E-14	8.07E-12
								Hcn4	9	2.366	1.943	3.845	2.46E-14	6.83E-12
								Dcc	18	2.530	1.484	2.797	2.21E-14	6.42E-12
								Tenm1	X	4.118	1.343	2.537	1.86E-14	5.51E-12
								Slc13a4	6	7.873	1.319	2.496	1.82E-14	5.45E-12
								Akap9	5	4.898	1.404	2.647	1.56E-14	4.77E-12
								Fn1	1	11.058	1.373	2.589	1.31E-14	4.22E-12
								Tenm3	8	10.203	1.490	2.808	1.20E-14	3.99E-12
								Myh11	16	2.926	1.585	2.999	1.11E-14	3.75E-12
								Tiam1	16	17.904	1.360	2.568	9.69E-15	3.35E-12
								Mdga1	17	7.328	1.633	3.101	8.06E-15	2.89E-12
								Ndst4	3	3.882	1.432	2.699	5.37E-15	2.07E-12
								Lgr5	10	2.097	3.629	12.372	5.34E-15	2.07E-12
								Col1a1	11	2.961	1.429	2.693	5.41E-15	2.07E-12
								Polr2a	11	15.921	1.361	2.568	4.94E-15	2.06E-12
								Igsf3	3	3.939	1.555	2.937	1.21E-15	5.97E-13
								Cspg4	9	3.433	1.324	2.503	8.14E-16	4.14E-13
								Scn5a	9	1.666	2.201	4.597	6.13E-16	3.60E-13
								Grm1	10	8.766	1.460	2.751	3.09E-16	1.88E-13
								Robo1	16	7.520	1.383	2.608	2.28E-16	1.44E-13
								Ngfr	11	2.361	2.550	5.857	1.54E-16	1.09E-13
								Ptpro	6	19.547	2.258	4.784	9.89E-17	7.24E-14
								Ptpn14	1	2.768	1.535	2.899	5.61E-17	4.67E-14
								Dlk1	12	3.946	1.864	3.641	3.73E-17	3.38E-14
								Eomes	9	3.592	7.127	139.807	3.78E-18	4.94E-15
								Filip1	9	6.238	1.633	3.102	3.00E-19	4.99E-16

								Vwf	6	3.821	1.816	3.520	2.24E-19	4.10E-16
								Nos1	5	11.660	1.634	3.104	2.09E-20	4.24E-17
								Nrp2	1	7.671	1.622	3.078	1.41E-20	3.23E-17
								Reln	5	10.356	1.876	3.670	6.63E-23	2.02E-19
								Gm28635	2	0.991	5.864	58.258	1.51E-24	5.52E-21
								Sv2c	13	6.719	2.018	4.051	1.44E-24	5.52E-21
